# N4BP1 is essential for the development of oral cancer via controlling both cancer cells and immune microenvironment

**DOI:** 10.1038/s41419-025-08229-0

**Published:** 2026-01-09

**Authors:** Yihua Song, Rong Sun, Jie Ji, Wen Zheng, Yanli Li, Xiaohong Guo, Liuting Chen, Yuanyuan Wu, Miaomiao Chen, Xingmei Feng, Mingbing Xiao, Renfang Mao, Yihui Fan

**Affiliations:** 1 Department of Pathogenic Biology, School of Medicine, Nantong University, Nantong, China; 2 Department of Stomatology, Affiliated Hospital of Nantong University, Medical School of Nantong University, Nantong, China; 3 Laboratory of Medical Science, School of Medicine, Nantong University, Nantong, China; 4 Department of Gastroenterology, Affiliated Hospital of Nantong University, Medical School of Nantong University, Nantong, China; 5 Department of Pathophysiology, School of Medicine, Nantong University, Nantong, China; 6 Jiangsu Province Key Laboratory in University for Inflammation and Molecular Drug Target, Nantong University, Nantong, China

**Keywords:** Oral cancer, Oral cancer

## Abstract

N4BP1 specifically degrades a subset of mRNA targets through their coding sequences and functions as a negative regulator of inflammation; however, its role in cancer development remains undefined. N4BP1 exhibits the highest expression in head and neck squamous cell carcinoma among all analyzed cancer types. Unlike wild-type mice, *N4bp1*^−/−^ mice did not develop visible tongue tumor masses in a 4-NQO-induced oral carcinogenesis model. Furthermore, *N4bp1*^−/−^ mice (86% vs 0%) exhibited significantly prolonged survival compared to wild-type mice within 26 weeks in 4-NQO-induced oral carcinogenesis model. Single-cell profiling demonstrated that N4BP1-deficient epithelial cells arrest at an early stage of cancerous transformation, while wild-type epithelial cells efficiently progress to an advanced stage of cancer. In established human cancer cell lines, N4BP1 also plays a crucial role in proliferation, migration, colony formation, and in vivo growth. Transcriptome profiling identified CCL2 and GM-CSF as downstream targets of N4BP1 in oral cancer. Apart from its intrinsic role in cancer cells, N4BP1-deficient cancer cells induce the differentiation of macrophages into the M1 phenotype. In N4BP1-deficient tissues, CCL2 and GM-CSF were significantly increased, accompanied by the accumulation of M1 macrophages and neutrophils. Our results demonstrate that N4BP1 is an essential gene in tongue cancer development. N4BP1 not only drives cancer cell evolution but also establishes an immune-suppressive microenvironment. N4BP1 is an endoribonuclease that specifically regulates a subset of mRNA targets (including CCL2 and GM-CSF) and plays an essential role in oral cancer.

## Introduction

Oral squamous cell carcinoma (OSCC) represents one of the most common subtypes of head and neck squamous cell carcinomas (HNSCC) [1]. Over the past several decades, survival rates for OSCC patients have remained relatively unchanged. Since its approval in 2006, Cetuximab—a monoclonal antibody targeting the epidermal growth factor receptor (EGFR)—has persisted as the only targeted therapy available for OSCC [2]. Whole exome sequencing conducted in OSCC has identified several recurrently mutated genes such as TP53, NOTCH1, CDKN2A, CASP8, HRAS, PI3KCA, and BRAF [3, 4]. Nonetheless, these cancer driver genes do not bring to targeted drugs for clinical treatment, and the molecular mechanism of OSCC needs to be further explored.

OSCC develops through a multistep process characterized by transformation of keratinocytes that consists of a disease spectrum from hyperplasia to metastatic disease [5, 6]. Throughout OSCC progression, both the transformed cells and their microenvironment are critical and mutually shaping to form malignant lesions [7]. For example, tumor cells secrete CCL2 (also called MCP-1) and TGFβ to attract and activate monocytes to generate angiogenic factors for tumor cell growth [8, 9]. OSCC produces the hematopoietic growth factors G-CSF and GM-CSF to stimulate the proliferation of neutrophil, macrophage, and keratinocyte [10, 11]. However, in certain contexts, CCL2 and GM-CSF can also promote anti-tumor immune responses [12]. Talimogene laherparepvec (T-VEC), a genetically modified herpes simplex virus type 1 (HSV-1), is designed to selectively lyse tumor cells and produce human granulocyte macrophage colony-stimulating factor (GM-CSF) for activation of antigen-presenting cells as well as induction of tumor-specific T-cell responses [13, 14]. Thus, the functions of secreting factors in cancer are highly microenvironment-dependent, and different combinations of these factors may lead to distinct biological outcomes. Exploring the regulation of secreting factors in tumor microenviroment not only provides new insights to understanding the mechanisms of oncogenesis, but also reveals novel strategies for cancer treatment.

Unlike exoribonucleases, endoribonucleases cleave RNA molecules internally and are well-documented to initiate RNA degradation in bacteria [15, 16]. Nevertheless, their roles in eukaryotes remain poorly understood. While the vast majority of eukaryotic endoribonucleases are involved in general RNA metabolism, a small subset acts specifically on subpopulations of cellular transcripts by recognizing particular sequences within the RNA substrates; these are referred to as context-specific endoribonucleases [17]. A typical representative among context-specific endoribonucleases is Regnase-1 (Regulatory RNase 1), also known as ZC3H12A or MCPIP-1. It harbors a PIN (PilT N terminus)-like RNase domain (also known as the NYN domain) with RNase activity and a CCCH-type ZF domain for RNA-binding [18, 19]. Regnase-1 is necessary for regulating immune cell activation and maintenance of immune homeostasis. It degrades mRNAs involved in immune cell activation and plays a critical role in many diseases [20, 21]. As a result, Regnase-1 becomes a valuable target that extensively tested in preclinical cancer models [22, 23]. These emerging evidences indicate that context-specific endoribonucleases not only have important functions in diseases but also represent promising druggable targets; however, the role of many of them in cancer is elusive.

N4BP1 is a context-specific endoribonuclease that contains an NYN domain and belongs to the family of Regnase-1-related endoribonucleases [18, 19]. Studies using N4BP1-deficient mice clearly demonstrated its critical roles in inflammation and autoimmune diseases [24–27]. A previous report indicates a potential role of N4BP1 in neuroblastoma by restricting tumor antigen presentation [28]. However, the role of N4BP1 in other cancers and its underlying mechanisms are unknown. Here, by using N4BP1-deficient mice, we demonstrated a pivotal role of N4BP1 in the development of oral squamous cell carcinoma. N4BP1 regulates multiple facets of cancer development, influencing not only cancer cells themselves but also the tumor microenvironment. These findings position N4BP1 as a potential druggable target for oral squamous cell carcinoma as well as other cancer types.

## Results

### N4BP1 has the highest level in head and neck squamous cell carcinomas (tongue squamous cell carcinoma)

**Fig. 1:**
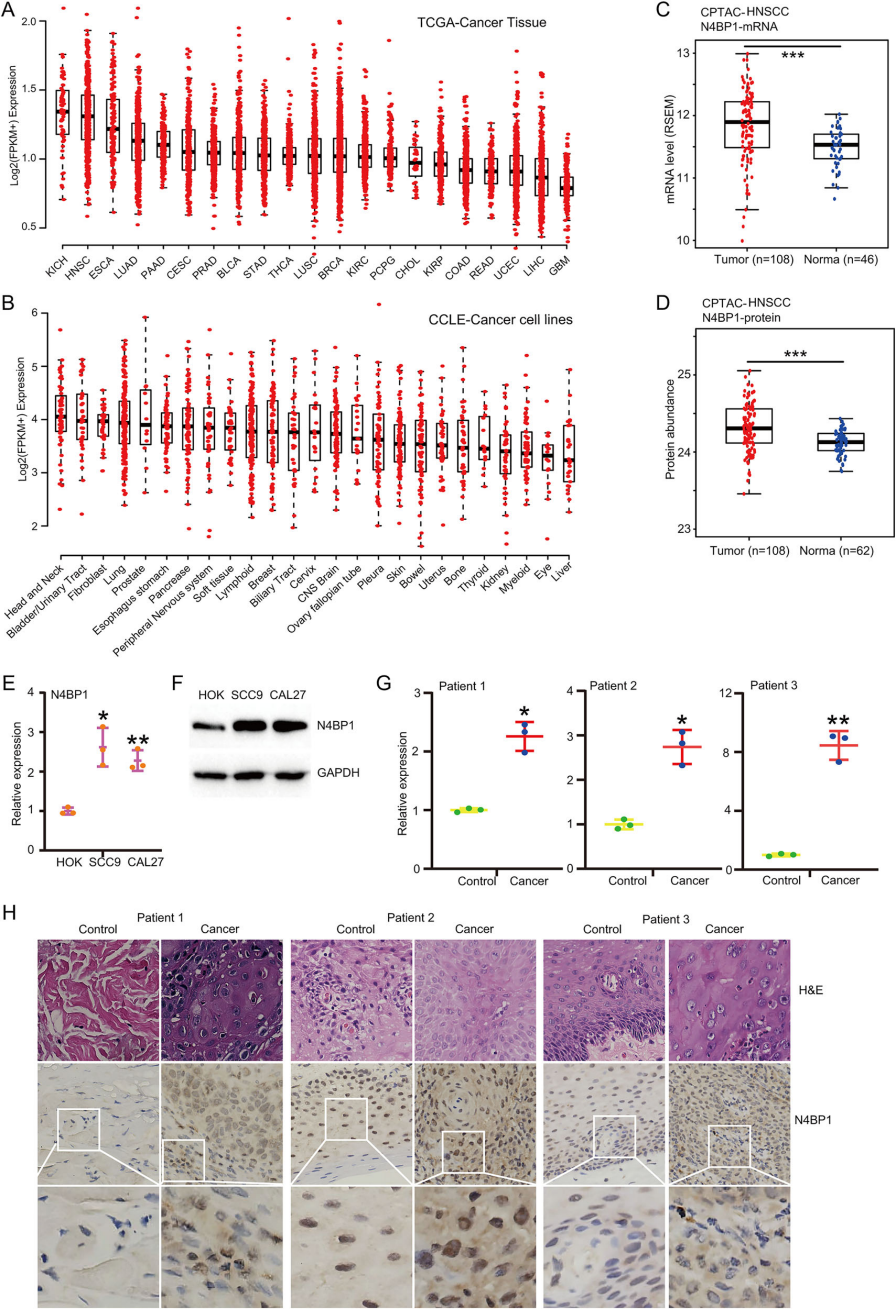
Increased expression of N4BP1 in squamous cell carcinoma of tongue is dependent on BRD4. **A** The raw data of RNA-seq from the Cancer Genome Atlas (TCGA) program were downloaded and reanalyzed (https://portal.gdc.cancer.gov/). The relative expression of N4BP1 in 21 cancer types was present; **B** the raw data of RNA-seq from Cancer Cell Line Encyclopedia (CCLE) were downloaded and reanalyzed (https://sites.broadinstitute.org/ccle/). The relative expression of N4BP1 in different cancer cell lines were present based on tissue type; **C** the mRNA data of head and neck squamous cell carcinoma from the Clinical Proteomic Tumor Analysis Consortium (CPTAC) were downloaded and reanalyzed; **D** the protein data of head and neck squamous cell carcinoma from the Clinical Proteomic Tumor Analysis Consortium (CPTAC) was downloaded and reanalyzed; **E** the mRNA level of N4BP1 in cell lines including HOK, SCC9 and CAL27 were examined by RT-PCR; **F** the protein level of N4BP1 in cell lines including HOK, SCC9 and CAL27 were examined by WB; **G** the total mRNA from three pairs of fresh tongue squamous cancer tissue and its non-neoplastic adjacent tissues were extracted and the mRNA level of N4BP1 was examined by RT-PCR; **H** the protein expression from three pairs of fresh tongue squamous cancer tissue and its non-neoplastic adjacent tissues were examined by IHC. Data in (**E**–**G**) are representative of three independent experiments. *p < 0.05; **p < 0.01; ***p < 0.001.

N4BP1 is a novel endoribonuclease that targets on mRNA coding sequences and plays a critical role in inflammatory response, but its role in cancer is unclear. To elucidate the potential role of N4BP1 in cancer, we conducted a comprehensive analysis of N4BP1 transcript levels across 21 different cancer types using data from the TCGA database. Among the analyzed tumor types, N4BP1 showed the highest expression in kidney chromophobe (KICH), head and neck squamous cell carcinoma (HNSCC), and esophageal carcinoma (ESCA) (Fig. 1A). To avoid tissue complexity, we also analyzed N4BP1 level in cancer cell lines by using the CCLE dataset (Fig. 1B). The results in cell lines are similar to those in cancer tissues, with higher N4BP1 expression observed in cell lines derived from head and neck cancers. To further explore the potential role of N4BP1 in HNSCC, we analyzed the mRNA and protein level of N4BP1 in HNSCC by using data from CPTAC. As shown, the level of N4BP1 was significantly upregulated in HNSCC compared to control tissues (Fig. 1C, D). Given that tongue (oral) squamous cell carcinoma is one of the most frequent subtypes in HNSCC, we examined the expression of N4BP1 in control human oral keratinocyte **(**HOK**)** cell line and two tongue squamous cell carcinoma cell lines (SCC9 and CAL27). When compared with control cells, an elevated level of N4BP1 was observed in tongue squamous carcinoma cells (Fig. 1E, F). Furthermore, the up-regulation of N4BP1 in tongue squamous carcinoma was validated in three tongue cancer tissues isolated from patients (Fig. 1G). Immunohistochemistry and western blotting also show the increased expression of N4BP1 in tongue cancer tissues compared to adjacent non-neoplastic tissues (Figs. 1H and S1A). At higher magnification, N4BP1 exhibits a dual localization in both nuclear and cytoplasmic compartments (Fig. 1H).

To understand the underlying mechanism of N4BP1 up-regulation in tongue cancers, we treated SCC9 and CAL27 cells with super-enhancer inhibitors. Super-enhancers are frequently implicated in driving oncogene overexpression in cancer. The increased N4BP1 was associated with super-enhancers, because super-enhancer inhibitor JQ-1, I-BET-762, and dBET6 significantly down-regulated the mRNA level of N4BP1 in SCC9 and CAL27 cells (Fig. S1B). Using western blot (WB) analysis, we further confirmed that inhibition of super-enhancers greatly reduced the expression of N4BP1 in both SCC9 and CAL27 cells (Fig. S1C). In summary, N4BP1 shows the highest expression in head and neck squamous cell carcinoma, with its level elevated in tongue cancer tissues relative to adjacent non-neoplastic tissues. Furthermore, the increased N4BP1 in tongue cancer is associated with super-enhancers.

### N4BP1-deficient mice are resistant to 4-NQO-induced squamous cell carcinoma of tongue

**Fig. 2:**
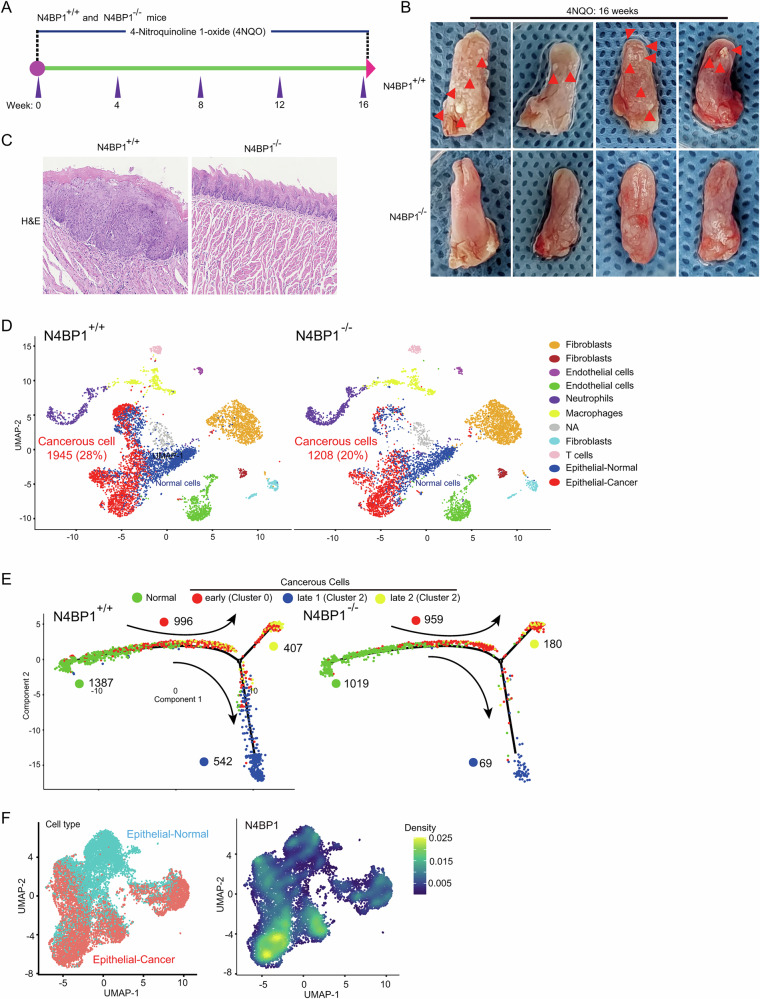
N4BP1-deficient mice are resistant to cancer development in chemical 4-NQO-induced cancer model. **A** Schematic presentation of 4-NQO-induced mice oral cancer model. Wild-type and N4BP1-deficient C57BL/6 mice were received 4-NQO (4-nitroquinoline-1-oxide) in drinking water for 16 consecutive weeks; **B** representative photos of 4-NQO-induced tongue squamous cancer; **C** H&E staining of 4-NQO-treated wild-type and N4BP1-deficient tongue tissues; **D** single-cell sequencing of 4-NQO-treated wild-type and N4BP1-deficient tongue tissues; We captured 34,497 cell from wild-type and 30,504 cells from *N4bp1*^−/−^ mice. Seven major transcriptional clusters, including fibroblasts, endothelial cells, neutrophils, macrophages, T cells, and epithelial cells, can be identified. The epithelial cells can be further divided into normal and cancerous cells based on copy number variation; **E** pseudotime analysis of normal and cancerous epithelial cells from 4-NQO-treated wild-type and N4BP1-deficient mice; **F** based on the single-cell-sequencing data from 4-NQO-treated wild-type and N4BP1-deficient tongue tissues, the level of N4BP1 in normal and cancerous cells were present. Data in (**B**, **C**) are representative of four independent experiments.

The increased level of N4BP1 in tongue squamous cell carcinoma (TSCC) suggests that it may play a crucial role in the progression of TSCC. To determine the role of N4BP1 in TSCC, we used 4-Nitroquinoline 1-oxide (4-NQO) to induce TSCC in mice (Fig. 2A). After 16 weeks of treatment, 4-NQO efficiently induced visible TSCC in all wild-type mice (n = 14, from four independent experiments) (Fig. 2B). In contrast, no visible tumors were observed in N4BP1 knockout mice (n = 14, from four independent experiments) (Figs. 2B and S2B). H&E staining confirmed the presence of tumor nodules in wild-type mice (Figs. 2C and S2C). In N4BP1 knockout mice, the epidermal tissue exhibited dysplasia but did not form tumor nodules (Figs. 2C and S2C). To further examine the critical role of N4BP1 in TSCC development, we conducted a 7-month survival analysis (16 weeks of 4-NQO treatment followed by 10 weeks of pure water) (Fig. S2A). All wild-type mice (100%, n = 7) died within 26 weeks after 16-week 4-NQO treatment period (Fig. S2D). In contrast, only one out of seven N4BP1^−/−^ mice (n = 7, 14%) died at week 26, and all other N4BP1^−/−^ mice still survived at week 28 (Fig. S2D). These results clearly demonstrate a critical role of N4BP1 in the development of TSCC.

To elucidate the cellular mechanisms of N4BP1 in TSCC, we conducted single-cell RNA-sequencing (scRNA-seq) analysis utilizing tongues from both wild-type and N4BP1 knockout mice after 16 weeks of 4-NQO treatment. We captured 34,497 cell from wild-type and 30,504 cells from *N4bp1*^−/−^ mice, respectively. Twenty percent of cells were randomly selected for downstream analysis. Using uniform manifold approximation and projection (UMAP), we identified seven major transcriptional clusters including fibroblasts, endothelial cells, neutrophils, macrophages, T cells, and epithelial cells (Fig. 2D). Within the epithelial population, cancerous and normal cells can be distinguished by copy number variation (Fig. 2D). Consistent with observed tumor nodules, both the number and proportion of cancerous cells were significantly reduced in N4BP1-deficient mice compared to wild-type mice (Fig. 2D). Among cancerous cells, there are three transcriptional clusters (clusters 0, 2, and 5). To understand the tumor progression under 4-NQO treatment, we performed pseudotime analysis of epithelial and cancerous cells. The trajectory indicated that cancer evolution initiated from normal epithelial cell, progressed to cancerous cluster 0, and then transitioned to either cancerous cluster 2 or 5 (Fig. 2E). Compared to wild-type cells, N4BP1-deficient cells were arrested predominantly at cancerous cluster 0 (Fig. 2E). In wild-type mice, over 60% of cancerous cells advanced to the final stages (clusters 2 and 5), whereas fewer than 5% of cancerous cells in N4BP1-deficient mice reached these stages (Fig. 2E). Furthermore, the level of N4BP1 in cancerous cells was significantly up-regulated when compared to normal cells (Fig. 2F). This observation is consistent with the increased N4BP1 level in human tongue squamous cell carcinoma (TSCC) compared to adjacent non-tumor tissues (Fig. 2F). Collectively, our in vivo model, coupled with single-cell analysis, unequivocally underscores the essential role of N4BP1 in facilitating the progression of 4-NQO-induced tongue cancer.

### N4BP1 controls multiple aspects of established human tongue squamous carcinoma cell lines

**Fig. 3:**
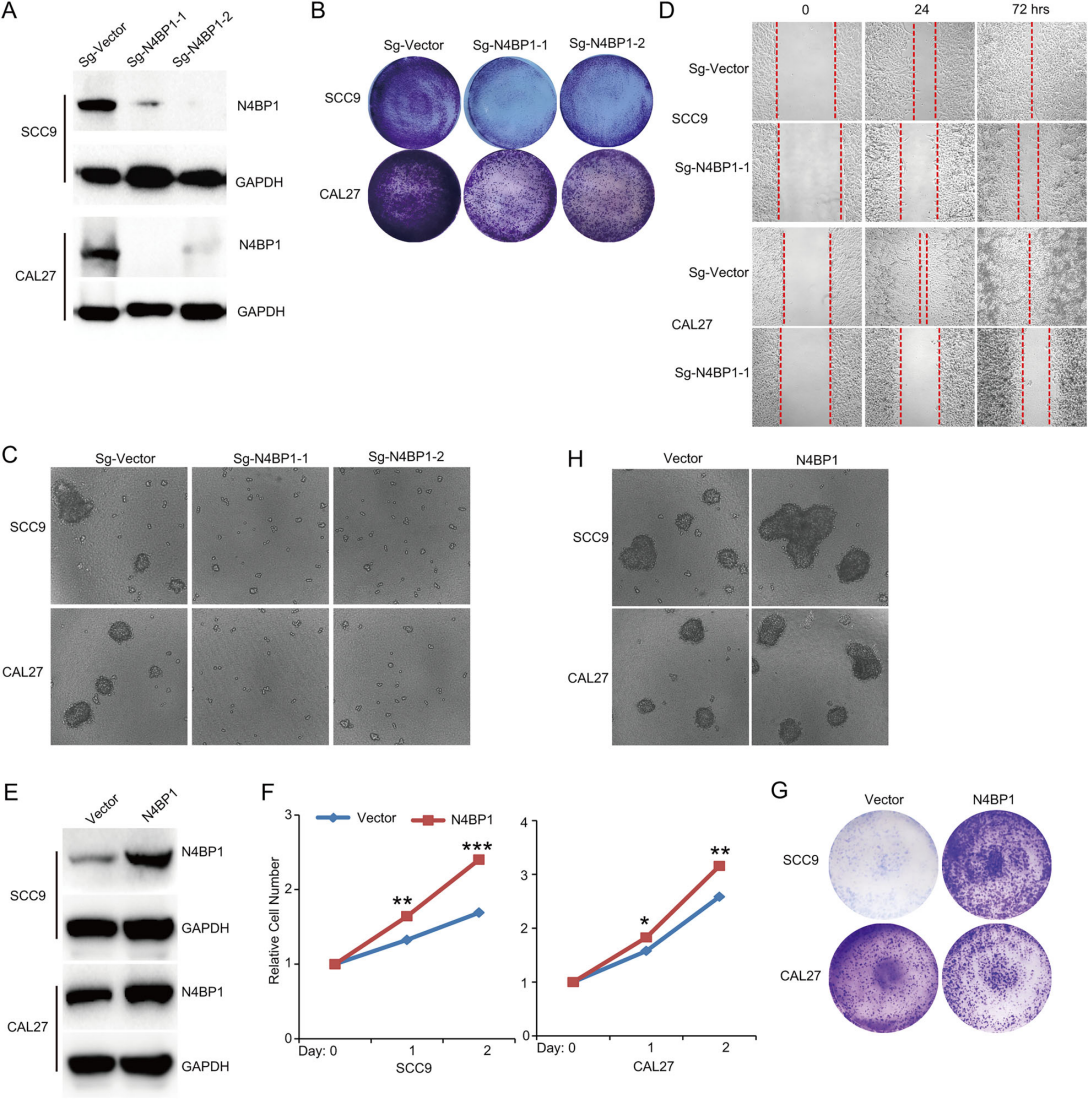
N4BP1 is critical in established human cancer cell lines. **A** Two sgRNAs targeted on N4BP1 were stably introduced in SCC9 and CAL27 cells. The protein level of N4BP1 was examined in control and N4BP1-deficient cells; **B** anchor-dependent colony formation assay was performed in control and N4BP1-deficient SCC9 and CAL27 cells; **C** anchor-independent colony formation assay was performed in control and N4BP1-deficient SCC9 and CAL27 cells; **D** scratch-healing assay was performed in control and N4BP1-deficient SCC9 and CAL27 cells; **E** SCC9 and CAL27 cells were transfected with plasmids encoding N4BP1 and selected by puromycin to establish stable cell lines. The total protein was extracted and examined by WB; **F** cell proliferation of N4BP1 ectopic expressed SCC9 and CAL27 cells was examined by CCK-8 assay; **G** anchor-dependent colony formation assay was performed in N4BP1 ectopic expressed SCC9 and CAL27 cells; **H** anchor-independent colony formation assay was performed in N4BP1 ectopic expressed SCC9 and CAL27 cells. Data in (**A**–**D**) are representative of three independent experiments. Data in (**E**–**H**) are representative of three independent experiments. *p < 0.05; **p < 0.01; ***p < 0.001.

Next, we examined whether N4BP1 also plays a critical role in established human tongue squamous carcinoma cells. We knocked out N4BP1 in SCC9 and CAL27 cells and confirmed the knockout by western blotting (Fig. 3A). In anchorage-dependent colony formation assay, the ability of N4BP1-deficient TSCC cells to form colonies was significantly impaired (Fig. 3B). Notably, in anchorage-independent colony formation assay, control cells formed large colonies but N4BP1-deficient TSCC cells failed to do so (Fig. 3C). In migration assays, N4BP1-deficient TSCC cells migrated slowly than control cells (Fig. 3D). Our results demonstrate a critical role of N4BP1 in human TSCC cells and N4BP1 contributes significantly in cell migration and colony formation. To further confirm the oncogenic role of N4BP1 in TSCC, we did ectopic expression of N4BP1 (Fig. 3E). Ectopic expression of N4BP1 enhanced cell proliferation (Fig. 3F). Ectopic expression of N4BP1 also promotes both anchorage-dependent and independent colony formation (Fig. 3G, H). Taken together, our results demonstrate that N4BP1 plays an essential role in TSCC cells and controls multiple aspects of their malignant behavior.

### N4BP1 is required for human TSCC growth in nude mice

**Fig. 4:**
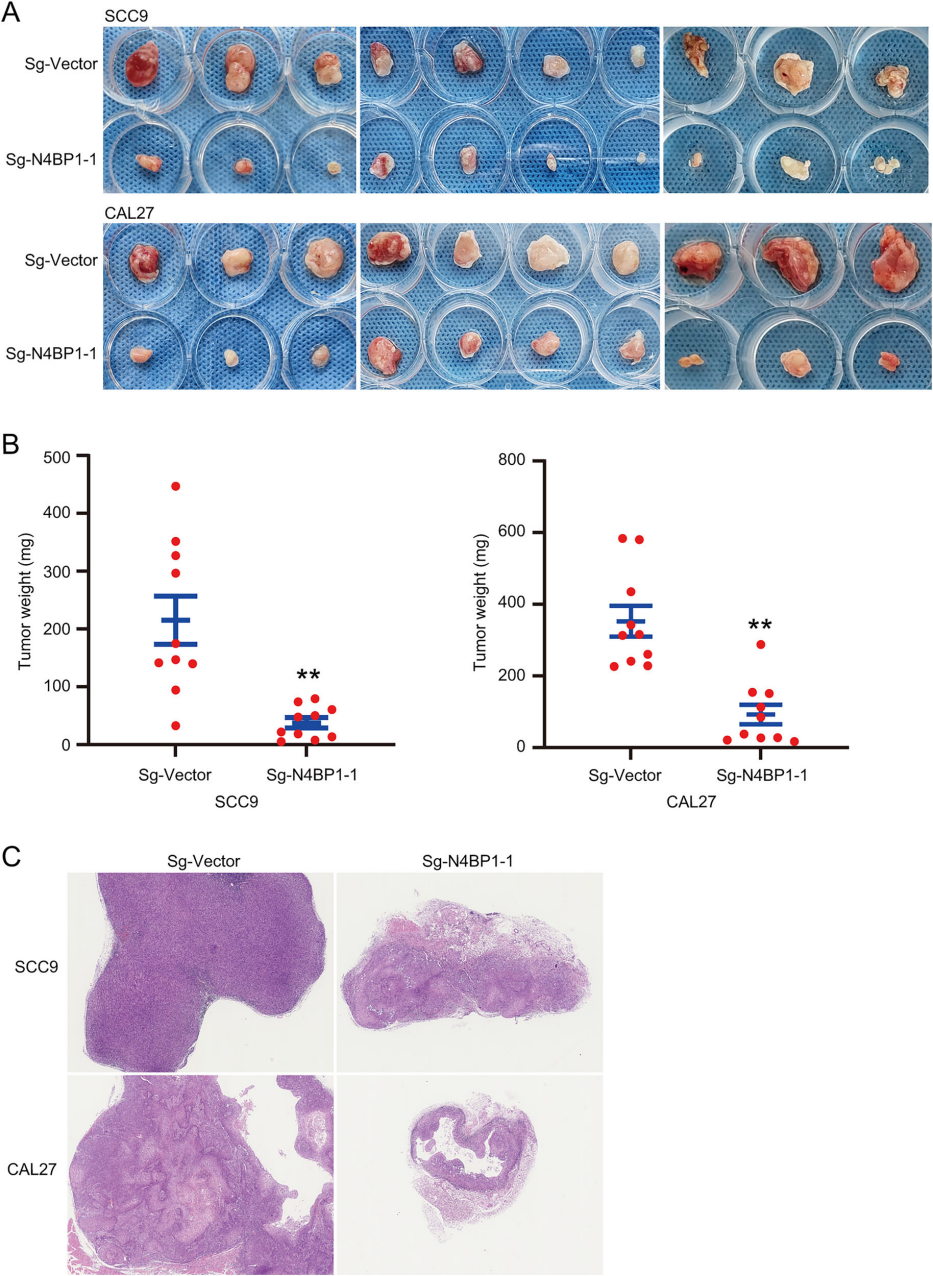
N4BP1 is important for tumor formation in nude mice. **A** N4BP1 knocking out SCC9 and CAL27 cells was injected subcutaneously. The growth of xenograft tumors in nude mice was monitored, and the representative images of isolated tumor mass were showed (n = 10 for each group); **B** tumor weight of subcutaneous tumors in each mouse were weighted and presented; **C** H&E staining was performed to determine the tumor tissues from subcutaneous tumors from N4BP1 knocking out SCC9 and CAL27 cells. Data in (**A**–**C**) are representative of three independent experiments. **p < 0.01.

The critical role of N4BP1 in human TSCC cells in vitro encouraged us to examine its function in vivo. We established TSCC xenografts in nude mice and found that both SCC9 and CAL27 cells could form tumors in vivo (Fig. 4A). Compared to control cells, N4BP1-deficient TSCC cells formed substantially smaller tumor masses (Fig. 4A). The weight of N4BP1-deficient tumors is much less than that of control tumors (Fig. 4B). H&E staining further confirmed that the nodules formed by N4BP1-deficient cells were markedly smaller than those from control cells (Fig. 4C). Collectively, our results clearly demonstrate the critical role of N4BP1 in the development of TSCC in vivo.

### RNA profiling identified N4BP1 downstream cytokines, including CCL2 and GM-CSF

**Fig. 5:**
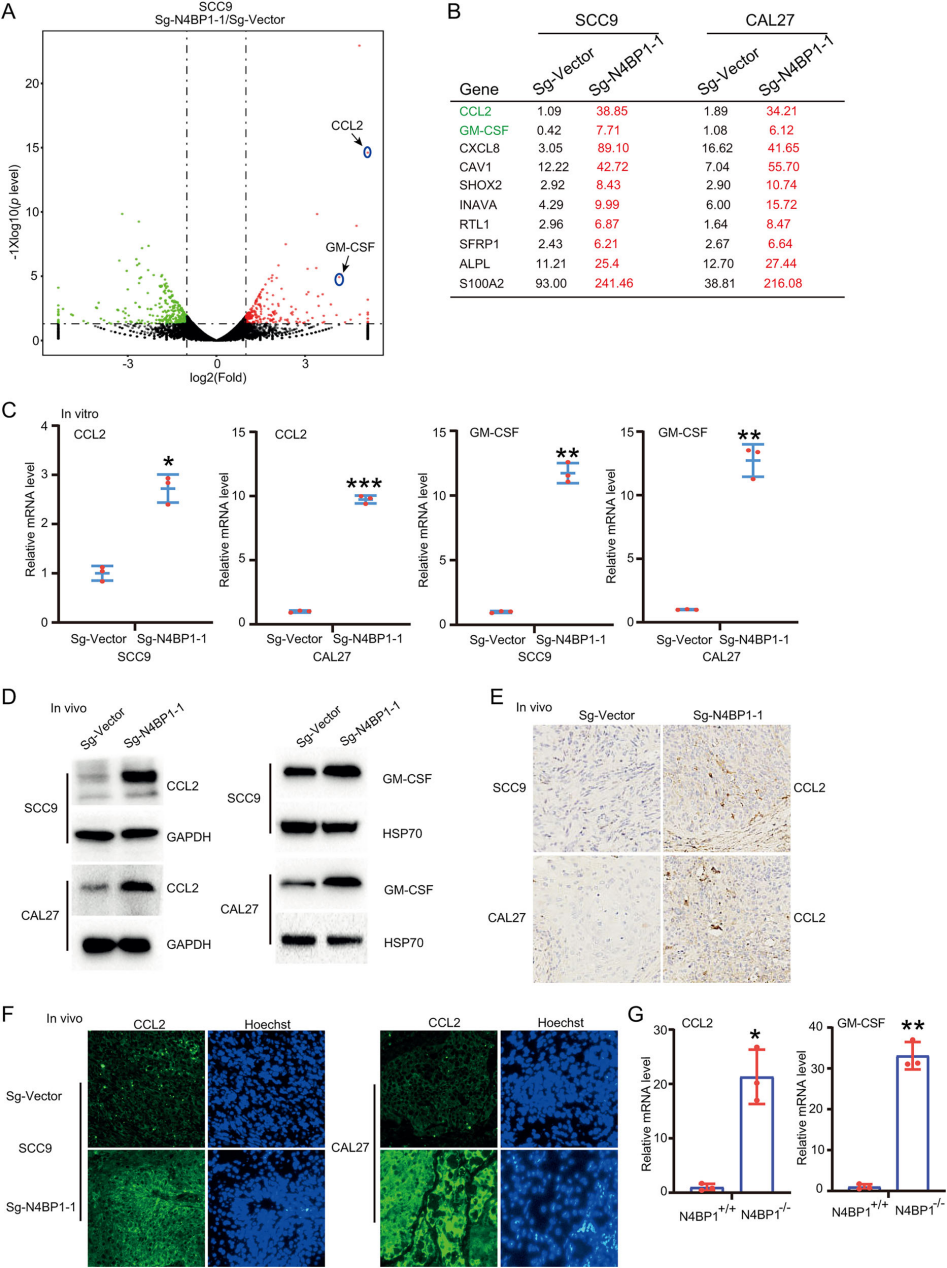
RNA profiling identify N4BP1 downstream cytokines, including CCL2 and GM-CSF. **A** The profiling of gene expression was performed by RNA-sequencing in SCC9 cells. The volcano plot was used to present the results and the expression of CCL2 and GM-CSF was labeled by arrows; **B** the 10 most up-regulated genes in N4BP1-deficient SCC9 and CAL27 cells were listed; **C** the mRNA level of CCL2 and GM-CSF was determined by real-time RT-PCR in N4BP1 wild-type and knockout SCC9 and CAL27 cells; **D** the protein level of CCL2 and GM-CSF in subcutaneous tumor from N4BP1 wild-type and knockout SCC9 and CAL27 cells was determined by WB; **E** the protein level of CCL2 in subcutaneous tumor from N4BP1 wild-type and knockout SCC9 and CAL27 cells was determined by IHC; **F** the protein level of CCL2 in subcutaneous tumor from N4BP1 wild-type and knockout SCC9 and CAL27 cells was determined by IF; **G** the mRNA level of CCL2 and GM-CSF in 4-NQO-treated wild-type and N4BP1-deficient tongue tissues was examined RT-PCR. Data in (**C**–**G**) are representative of three independent experiments. *p < 0.05; **p < 0.01; ***p < 0.001.

To elucidate the molecular mechanism by which N4BP1 modulates TSCC, we conducted RNA profiling on control and N4BP1-deficient TSCC cells. Given that N4BP1 is an endoribonuclease that specifically targets mRNA coding sequences, we focused on genes that were up-regulated following N4BP1 knockout. In SCC9 cells, we identified 100 up-regulated genes (fold change>2, expression level>5, and P < 0.05) in sg-N4BP1 cells (Fig. 5A). Similarly, in CAL27 cells, we identified 99 up-regulated genes (fold change>2, expression level>5, and P < 0.05) in sg-N4BP1 cells (Fig. S3A). The EMT-related genes are not significantly affected by knocking out N4BP1 (Fig. S3B). The ten most up-regulated genes upon N4BP1 deletion in both cell lines were listed in Fig. 5B. Among these, CCL2 and GM-CSF (CSF2) were two of the most significantly up-regulated genes upon N4BP1 knocking out. To confirm the up-regulation of CCL2 and GM-CSF following N4BP1 knockout, we used RT-PCR to examine their expression in control and N4BP1-deficient HSCC cells (Fig. 5C). Consistently, the mRNA levels of CCL2 and GM-CSF were significantly up-regulated in N4BP1-deficient cells under in vitro culture conditions (Fig. 5C). The protein levels of CCL2 and GM-CSF were also significantly increased in N4BP1-deficient HSCC cells (Fig. S3C). In tumor tissues established in vivo, the levels of CCL2 and GM-CSF were also significantly up-regulated in N4BP1-deficient tumors compared to controls, as examined by RT-PCR, western blotting (WB), immunohistochemistry (IHC), and immunofluorescence (IF) (Figs. 5D–F and S3D). To further explore the role of CCL2 and GM-CSF in TSCC, we analyzed their expression in 4-NQO-treated tongue from wild-type and N4BP1 knockout mice (Fig. 5G). The expression of CCL2 and GM-CSF in N4BP1 knockout mice was significantly up-regulated compared to it in wild-type mice after 16-week treatment with 4-NQO (Figs. 5G and S3E). Immunofluorescence and IHC analysis also revealed the increased expression of CCL2 and GM-CSF in N4BP1-deficient mice (Fig. S3F, G). Our results demonstrated that CCL2 and GM-CSF are significantly up-regulated upon deletion of N4BP1, both in vitro and in vivo.

In addition to CCL2 and GM-CSF, we also tested other potential N4BP1 targets listed in Fig. 5B. As shown, N4BP1 also significantly inhibits the level of CXCL8 at both mRNA and protein levels (Fig. S4A–C). Given that CCL2, GM-CSF, and CXCL8 primarily function within the tumor microenvironment, we expected that other N4BP1 targets might directly affect cancer cells. One of interesting targets in Fig. 5B is S100A2. Overexpression of N4BP1 strongly inhibited both the mRNA and protein expression of S100A2 (Fig. S4D–F). To examine the potential role of S100A2 in human HNSCC, we overexpressed it in SCC9 and CAL27 cells (Fig. S4G, H). Interestingly, overexpression of S100A2 could significantly inhibit migration (Fig. S4I). Our results suggest that N4BP1 controls multiple targets, such as CCL2, GM-CSF, CXCL8, and S100A2, to regulate multiple tumor malignant behaviors. However, we will focus on CCL2 and GM-CSF in this manuscript and demonstrate their role in tumor microenvironment.

### N4BP1 degrades mRNAs encoding CCL2/GM-CSF, and its inhibition promotes macrophage differentiation towards the M1 phenotype

**Fig. 6:**
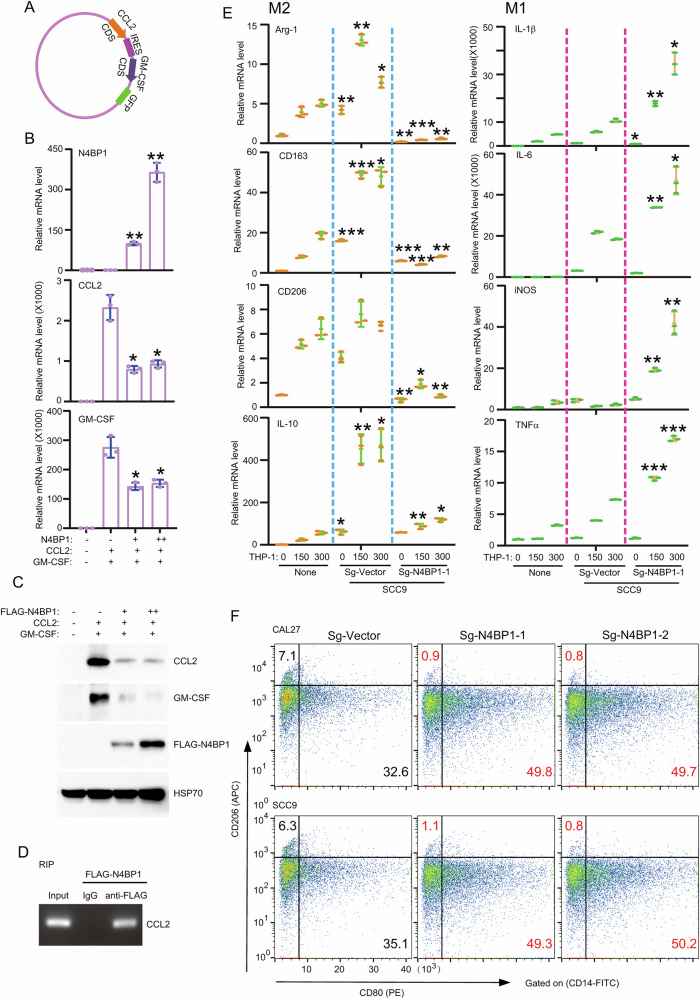
N4BP1 degrades CCL2 and GM-CSF to promote macrophage differentiate to M2 phenotype. **A** Schematic diagram to show the plasmid encoding coding sequences of CCL2 and GM-CSF; **B** 293T cells were transfected with plasmids containing CDS from CCL2 and GM-CSF with different amount of plasmids encoding N4BP1. The mRNA level was determined by RT-PCR; **C** 293T cells were transfected with plasmids containing CDS from CCL2 and GM-CSF with different amount of plasmids encoding N4BP1. The protein level was determined by WB; **D** plasmids encoding FLAG-N4BP1 were transfected in 293T cells and immunoprecipitated by anti-FLAG antibody. The association between FLAG-N4BP1 and CCL2 mRNA was examined by RNA immunoprecipitation; **E** THP-1 cells were activated by PMA before co-cultured with supernatant from control and N4BP1-deficient SCC9 and CAL27 cells. The M2 markers, including Arg-1, CD163, CD206, and IL-10, were examined by RT-PCR. The M1 markers, including IL-1β, IL-6, iNOS, and TNF-α, were examined by RT-PCR; **F** activated THP-1 cells were cultured with supernatant from control and N4BP1-deficient SCC9 and CAL27 cells. Cells were stained by CD14-FITC, CD80-PE, and CD206-APC and analyzed by FACS. Shown are representative plots from one of three independent experiments. Data in (**B**, **C**, **E**, **F**) are representative of three independent experiments. *p < 0.05; **p < 0.01; ***p < 0.001.

To further explore whether CCL2 and GM-CSF are direct targets of N4BP1, we cloned their coding sequences (Fig. 6A). Overexpression of N4BP1 significantly degrades the transcripts of CCL2 and GM-CSF (Fig. 6B). Overexpression of N4BP1 also significantly inhibits protein level of CCL2 and GM-CSF (Fig. 6C). RNA immunoprecipitation assays revealed that N4BP1 binds with CCL2 mRNA (Fig. 6D). Thus, our results demonstrated that CCL2 and GM-CSF are direct targets of N4BP1 in TSCC. Due to the critical role of CCL2 and GM-CSF in macrophage recruitment and differentiation, we culture control and N4BP1-deficient tumor cell with activated THP-1 cells. As shown, M1 macrophage markers-including IL-1β, IL-6, iNOS, and TNFα-were significantly increased in THP-1 cells co-cultured with N4BP1-deficient TSCC cells compared to those co-cultured with control tumor cells (Fig. 6E). However, M2 macrophage markers-including Arg-1, CD163, CD206, and IL-10-were down-regulated in THP-1 cells co-cultured with N4BP1-deficient cells relative to controls (Fig. 6E). Using supernatants from N4BP1-deficient TSCC or control cells, we obtained similar results (Fig. S5). To further confirm this conclusion, we used flow cytometry to analyze M1 or M2 macrophage polarization in the co-culture system. Again, macrophages co-cultured with N4BP1-deficient TSCC cells are favor to M1 differentiation, but these co-cultured with control TSCC cells are favor to M2 differentiation (Fig. 6F). Thus, our results demonstrated that N4BP1 directly degrades downstream targets such as CCL2 and GM-CSF to promote macrophage differentiate to M2 phenotype.

### N4BP1 established a tumor-suppressive microenvironment by inhibiting macrophage M1 differentiation and excluding neutrophils

**Fig. 7:**
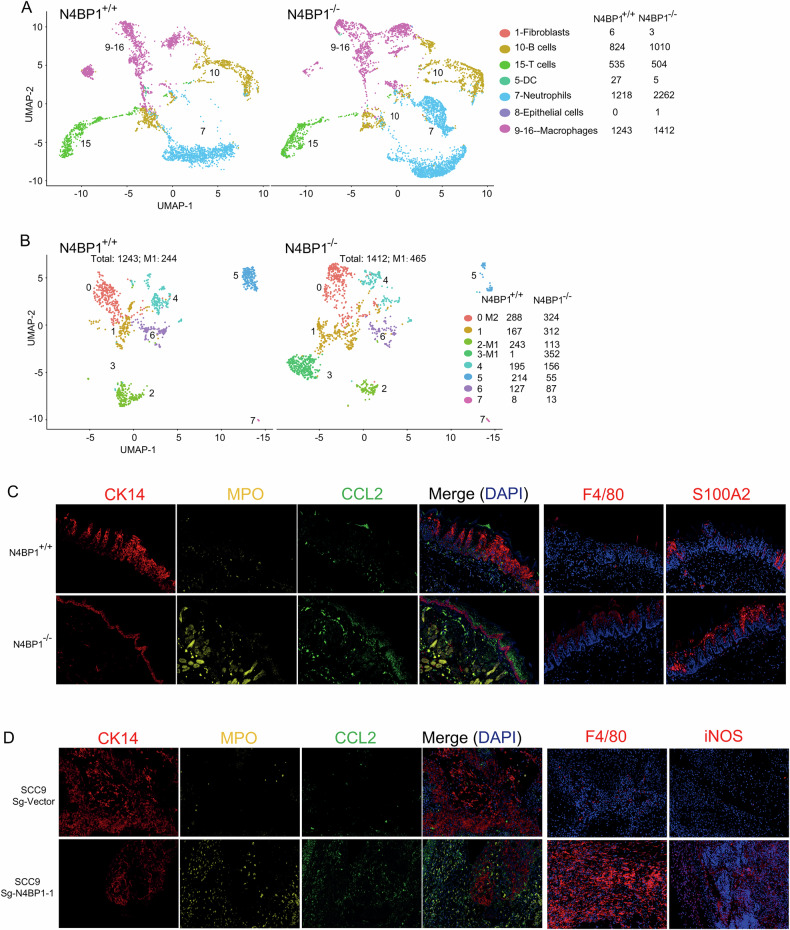
Elevated N4BP1 established a tumor-suppressive microenvironment by promoting macrophage M2 differentiation and inhibiting recruitment of neutrophils. **A** Single-cell sequencing of 4-NQO-treated wild-type and N4BP1-deficient tongue tissues. Immune cells, including B cells, T cells, DC, neutrophils, and macrophages are identified; **B** single-cell sequencing of 4-NQO-treated wild-type and N4BP1-deficient tongue tissues. The macrophages were identified and further analyzed to distinguish M1 and M2 phenotypes; **C** multiplex immunohistochemistry was performed in 4-NQO-treated wild-type and N4BP1-deficient tongue tissues. The markers, including CK14, MPO, CCL2, F4/80, and S100A2 were examined; **D** multiplex immunohistochemistry was performed in tumor tissues from sg-Vector and sg-N4BP1 SCC9 cells. The markers, including CK14, MPO, CCL2, F4/80, and iNOS were examined.

To further characterize the immune microenvironment in wild-type versus N4BP1-deficient tumors, we analyzed our single-cell sequencing data and found that the overall number and percentage of immune cells—including T cells, B cells, and macrophages—were comparable between wild-type and N4BP1-deficient mice (Fig. 7A). However, upon closer examination of macrophage subsets, we observed that the proportion of M1 macrophages was significantly higher in N4BP1-deficient mice than in wild-type mice (Fig. 7B). These results suggest that the microenvironment in wild-type tumor was more favorable to M2 macrophage differentiation. These results demonstrated that N4BP1 in TSCC cells shaped their secretion factors to promote macrophage differentiate to M2 phenotype. To further examine the relationship between CCL2/GM-CSF and macrophage differentiation, we conducted multiplex immunohistochemical analysis to investigate the role of CCL2 and GM-CSF in tumor microenvironment in vivo. In tongue tissues from N4BP1-deficient mice, the increased level of CCL2 was associated with increased macrophage marker F4/80 (Fig. 7C). In N4BP1-deficient mice, the level of S100A2 was also increased (Fig. 7C). Consistently, N4BP1-deficient SCC9 and CAL27 tumor tissues exhibited increased CCL2 expression along with increased M1 macrophage marker, iNOS (Figs. 7D and S6). Thus, our results demonstrated that CCL2 and GM-CSF are key downstream targets of N4BP1 for establishment tumor-suppressive immune microenvironment.

Beside increased M1 macrophage, we also observed a significant increase in the number of neutrophils in N4BP1-deficient tissues (Fig. 7A). In our analysis, neutrophils can be clustered into two distinct subgroups (Fig. 7A). One cluster of neutrophils is uniquely present in N4BP1-deficient tissue (Fig. 7A). To further elucidate the functional relevance of CCL2, GM-CSF and CXCL8, we analyzed their corresponding receptors across different immune cells. Consistently, macrophages show higher level of CCR2 and CSF2RB (Fig. S7). Neutrophils were enriched with receptors of CSF2RB and CXCR2 (Fig. S7). In parallel, multiplex immunohistochemical analysis revealed that the level of neutrophil marker (MPO) was elevated in tongue tissues from N4BP1-deficient mice, and in N4BP1-depleted SCC9 and CAL27 xenograft tumors. A finding that was concomitant with the enrichment of the CSF2RB and CXCR2 receptors in neutrophils (Figs. 7C, D and S7). Taken together, our results demonstrate a critical role of CCL2, GM-CSF, and CXCL8 in the establishment of an immune-suppressive tumor microenvironment.

## Discussion

Head and neck cancer encompasses various cancers originating from diverse locations within the mucosal linings of the upper aerodigestive, but most of them commonly originated from squamous cells and are thought to share common carcinogenic pathways during carcinogenesis [29–31]. Tongue squamous cell carcinoma, a prevalent form of HNSCC, originates from squamous cells and shares molecular mechanisms with other HNSCCs. The carcinogenesis of tongue squamous cell carcinoma is a highly complex, multifactorial process that occurs when epithelial cells are transformed from hyperplasia to dysplasia and eventually to invasive SCC [30, 32]. During the epithelial transformation process, the microenvironment is also adapted to immunosuppressive state [33]. Here, we found that N4BP1 is critical for epithelial cells transformation to squamous cell carcinoma. Upon 4-NQO induction, only very few N4BP1-deficient epithelial cells are transformed to carcinoma, and most of them are blocked at dysplasia stage. Furthermore, in N4BP-deficient tongue tissue, the microenvironment remained immunoactive. Our results clearly demonstrate a central role of N4BP1 in both epithelial transformation and the establishment of an immunosuppressive microenvironment.

Only a few epithelial cells that accumulate genetic, epigenetic, and external driver events eventually undergo transformation [34, 35]. During the transformation process, appropriate oncogenic functions of key proteins may be necessary for the cells to progress successfully to the next stage. By analyzing our single-cell sequencing data, we found four distinct populations of epithelial cells, including one normal population and three cancerous subtypes. The developmental trajectory proceeds from normal epithelial cells to cluster 0, and then further advances to either cluster 2 or 5 (Fig. 2E). In wild-type mice, approximately half of the cancerous cells progress to late stages (clusters 2 or 5), indicating successful malignant transformation. However, very few N4BP1-deficient epithelial cells reach to late stages, with the majority arrested at early cancerous state (cluster 0). These results suggest that the function of N4BP1 is required for transmission from early to late stages of carcinogenesis. N4BP1 may facilitate this progression by helping early-stage cancerous cells suppress key checkpoint proteins such as S100A2, thereby overcoming barriers in developing into late-stage cancerous cells. S100A2 is a potential tumor suppressor in multiple cancer types, but its role remains under debate [36, 37]. S100A2 has also been reported to play an inhibitory role in the development of oral cancers [38, 39]. In our results, we found that S100A2 is a downstream target of N4BP1 and its overexpression inhibits migration (Fig. S4D–I). However, whether S100A2 is the central player downstream of N4BP1 in cancer cells remains to be explored.

In addition to the transformation of cancerous cells, the adaptation of microenvironment from immune activation to immune suppression is also essential for cancer development. One important event in this process is the change of macrophage from the M1 (pro-inflammatory) to the M2 (anti-inflammatory). In our single-cell analysis comparing wild-type and N4BP1 knockout mice, we found eight distinct macrophage populations (Fig. 7B). Among them, one population (cluster 0) was identified as M2 macrophages, while two populations (clusters 2 and 3) were identified as M1 macrophages. In N4BP1-deficient mice, the number of M1 macrophages was significantly higher than in wild-type mice. More importantly, we found that the cluster 3 M1 macrophages were uniquely present in N4BP1-deficient mice. We also found that the number of neurtrophils in N4BP1-deficient mice was also significantly higher than in wild-type mice (Fig. 7A). Based on our data, neutrophil can be subdivided into two subpopulations, one of which was uniquely in N4BP1-deficient mice. The unique presence of both M1 macrophages and a neutrophil subpopulation in N4BP1-deficient mice was clearly observed. But their roles in cancer remain to be clearly defined. By mRNA profiling, we found that these cytokines, including CCL2, GM-CSF, and CXCL8, might be important downstream targets of N4BP1 in establishing the cancer microenvironment.

During cancer development, cancer cells may utilize RNA-binding proteins (RBPs) to reversibly and quickly regulate gene transcripts for fast-changing microenvironment, but the role of RBP in cancer is less explored [40]. It was recently reported that FMRP does not impact tumor cell growth but facilitates immune evasion by controlling CCL7, IL-33, and PROS1 [41]. Among endoribonucleases, only a few of them are very specifically targeting on a subset of mRNAs that are called sequence-specific endoribonucleases. N4BP1 is one such sequence-specific endoribonuclease that specifically recognizes mRNA coding sequences (CDS) [42]. These sequence-specific endoribonucleases could precisely control a few mRNA targets for biological processes. However, their role in cancer development and personalized therapy is poorly defined. Here, we reported that N4BP1, a sequence-specific endoribonuclease, plays a very important role in both tumor cell growth and immune evasion. These sequence-specific endoribonucleases might be important for cancer cells adapt to the tumor microenvironment. Notably, the N4BP1-deficient mice develop and grow normally, suggesting that disruption of N4BP1 may significantly impede cancer development while having minimal adverse effects on patients.

## Conclusions

In summary, our study utilizing a genetic mouse model and single-cell sequencing has elucidated the critical role of N4BP1 in the progression of tongue squamous cell carcinoma. N4BP1 not only broadly affects cancer cells but also controls the tumor microenvironment. In contrast to Regnase-1—another member of N4BP1 family that commonly inhibits cancer development, N4BP1 promotes cancer development. N4BP1 is a sequence-specific endoribonuclease that involved in multiple aspects during cancer development. Due to the critical role of N4BP1 in oral squamous cell carcinoma, it might be a very promising druggable target for treatment.

## Material and methods

### Experimental animals, cell lines, and patient tissue samples

The SPF-grade BALB/c-nu athymic nude mice (8–10 weeks of age, weighing 18–24 g) and *N4bp1*^+/+^ and *N4bp1*^−/−^ C57BL/6 mice (8–10 weeks of age, weighing 18–24 g) were provided by the Experimental Animal Center of Nantong University. The HOK, SCC9, and CAL27 cells were donated by the Department of Stomatology of the Affiliated Hospital of Nantong University, and the THP-1 cells were donated by the Clinical Laboratory Center of the Affiliated Hospital of Nantong University. All cells were cultured in DMEM (high glucose) complete medium, which contained 10% fetal bovine serum, 1% streptomycin, and penicillin. All cells were statically cultivated in an incubator with 5% CO_2_ and a constant temperature of 37 °C. All cells requiring suspension culture were cultured in RPMI-1640 complete medium, which contained 10% fetal bovine serum, 1% streptomycin, and penicillin. All the cancer tissues of the OSCC patients were obtained with the informed consent of the patients and were in compliance with the requirements of the Medical Ethics Committee of the Affiliated Hospital of Nantong University. All mice were housed in a pathogen-free environment and handled according to protocols approved by animal Ethics Committee of Nantong University.

### Bioinformatics analysis

The Cancer Genome Atlas (TCGA) database (https://portal.gdc.cancer.gov/) is the most extensively applied database in cancer research, mainly storing genomic data of various tumors. In this study, the TCGA database was utilized to download the expression data of N4BP1 gene in various human cancers, and each expression value was subjected to conversion analysis. The Cancer Cell Line Encyclopedia (CCLE) database (https://portals.broadinstitute.org/ccle) is a large publicly accessible tumor genomic database over 1000 tumor cell lines. Through the utilization of this database, we obtained the expression of the N4BP1 gene in these tumor cell lines. The Clinical Proteomic Tumor Analysis Consortium (CPTAC) database (https://cptac-data-portal.georgetown.edu/datasets) compiles genomic and proteomic data related to human HNSCC. Through this database, we analyzed the expression level of N4BP1 mRNA in 108 HNSCC tumor tissues and 46 normal tissues, as well as the expression level of N4BP1 protein in 108 HNSCC tumor tissues and 62 normal tissues.

### Plasmid construction and establishment of stable cell lines

The sgRNA and overexpression plasmids were contructed as previously reported. All plasmids were validated through direct sequencing. One day prior to transfection, cells were digested with trypsin and collected by centrifugation. The pellet was mixed with complete medium and plated onto 6-well plates to ensure a cell density of approximately 70%. Before transfection, the medium was replaced with serum-free medium. The transfection efficiency was observed using a fluorescence microscope. Puromycin was used for selection. The knockdown and overexpression efficiency was validated by WB analysis.

### CCK-8 assay

Cells were digested using trypsin to prepare the cell suspension, which was then inoculated into 96-well plates at 100 μL per well, with no less than 3,000 cells per well. The cells were cultivated overnight or for a certain period as required by the experiment. At the designated time point, the culture medium was removed, and the wells were washed twice with PBS. 100 μL of CCK-8 solution (CCK-8 stock solution: serum-free RPMI medium = 1:9) was added to each well under dark conditions and with care to avoid bubbles. Incubation was continued in the incubator for 1–4 h. Finally, the absorbance was measured at 450 nm using a microplate reader.

### Cloning formation experiment

The cells were digested using trypsin to prepare a single-cell suspension. The cells were counted with a hemocytometer, and the cell concentration was adjusted to an appropriate density. The cell suspension was inoculated into 6-well plates, an appropriate amount of culture medium was added, and the plates were placed in a culture incubator with 5% CO_2_ at 37 °C for cultivation. The culture medium was replaced every 2–3 days. After 10-14 days of cultivation, the cell culture medium was aspirated and washed once with PBS. The samples were fixed with 4% paraformaldehyde solution for 20 min, removed and washed three times with PBS. The samples were stained with crystal violet staining solution for 30 min, the staining solution was aspirated, and the samples were washed three times with PBS. Photos were taken to record the staining results.

### Spheroid formation assay

The agarose powder was mixed with sterile water until complete dissolution, followed by autoclaving and cooling for subsequent use. 20 μL of 1% agarose solution was taken to coat the bottom of the 96-well plate to form a thin film, which was left to solidify. Subsequently, 75 μL of warm 1% agarose solution was added to the wells to form a concave surface, and 200 μL of sterile water was filled in the outermost wells. The agarose was allowed to fully solidify after cooling for 1 h. The cells were digested using trypsin to prepare a single-cell suspension. After counting with a hemocytometer, 5 × 10^3^ cells were added to each well, with a total volume of 200 μL. The plates were placed in the incubator for culture, and the cell growth was inspected and recorded regularly. The medium was changed after 4 days by extracting the old medium and adding an equal volume of fresh medium. On the 7th day, the growth of the tumor spheroids was observed and photographed using a microscope.

### Cell scratch experiment

Under sterile conditions, cells were inoculated into 6-well culture plates. Once the cells reached a certain density, a distinct scratch was made on the cell surface using a sterilized pipette tip, with a moderate scratch depth for observing cell migration. The scratch area was gently rinsed with PBS to remove impurities. After rinsing, 4% paraformaldehyde could be selected for use to fix the cells for better observation of the migration behavior. The cell migration at different time points was recorded and photographed under a microscope for the analysis of the cell migration dynamics.

### Subcutaneous tumor model in nude mice

Our study examined male mice because male animals exhibited less variability in phenotype. Male BALB/c-nu mice aged 6–8 weeks and weighing 18–24 g were divided into two groups. One group was inoculated with SCC9 cells, and the other with CAL27 cells to establish subcutaneous tumor models in nude mice, with 10 mice in each group. The control group (Sg-Vector) and the N4BP1 knockout group (Sg-N4BP1-1) cells were injected subcutaneously on both axillae, respectively. The tumor growth was observed regularly. At the 16th week, the mice were anesthetized with 35–40 mg/kg of 1% pentobarbital sodium by intraperitoneal injection and then sacrificed by cervical dislocation. After dissection, the transplanted tumors were removed and weighed for recording.

### Mouse model of tongue cancer induced by carcinogens

Our study examined male mice because male animals exhibited less variability in phenotype. Male C57BL/6 mice aged 6–8 weeks and weighing 18–24 g were divided into two groups. One group was wild-type C57BL/6 mice, and the other was N4BP1 gene knockout C57BL/6 mice, with 10 mice in each group. The 4-NQO reagent was dissolved in sterilized tap water to prepare a 0.001% 4-NQO drinking solution for feeding *N4bp1*^+/+^ and *N4bp1*^−/−^ mice for 16 weeks. The general behavior, weight changes, and signs of poisoning or disease of the mice were constantly observed. At the 16th week or when the animal’s weight dropped by more than 20%, the mice were euthanized by cervical dislocation after intraperitoneal anesthesia with 35–40 mg/kg of 1% pentobarbital sodium. The mice were dissected, and the tongue tissue was collected immediately.

### Preparation of tissue specimen paraffin blocks

Biological tissue samples were collected and classified, and labeled. The samples were placed in 4 mL EP tubes and fixed with formaldehyde or formalin solution to prevent spoilage and autolysis. Then, dehydration treatment was carried out by gradually replacing with ethanol of higher concentrations, ensuring the complete immersion of the tissues and avoiding drying and shrinking. The dehydrated tissues were placed in molds, molten paraffin was added, and they were placed on a pre-cooled paraffin embedding machine. The embedded wax blocks were cut into thin sections of 3–5 μm thickness and placed on glass slides and dried using a baking machine for subsequent experiments.

### Hematoxylin and eosin (H&E) staining

The tissue sections were dewaxed in xylene for 5–10 min successively, and then soaked in absolute ethanol, 90% alcohol, 80% alcohol, and 70% alcohol for 2 min respectively, and finally rinsed with clear water. Subsequently, the sections were stained in hematoxylin solution for 8–15 min, and the staining solution was rinsed off with clear water. Then, the sections were treated in the differentiating solution for several seconds to 30 s, and rinsed with clear water for bluing. After that, the sections were stained in eosin solution for 2–5 min, and soaked in 75% alcohol, 95% alcohol, and absolute ethanol for 30 s, respectively, followed by transparency in xylene for 1 min. Finally, the sections were mounted with neutral gum and observed under a microscope.

### Immunohistochemical staining

The tissue sections were placed in a pressure cooker, and citrate repair buffer was added to ensure complete immersion. The mixture was heated to the appropriate pressure. After cooling, the sections were rinsed with PBS to remove the repair solution. The sections were immersed in 3% H_2_O_2_ and incubated at room temperature to block endogenous peroxidases. The primary and secondary antibodies were selected based on the experimental requirements, diluted respectively, and dropped onto the sections. They were incubated at 4 °C or at room temperature overnight or for 2 h. The sections were rinsed with PBS to remove the residual antibodies. The DAB chromogenic solution was prepared and dropped onto the sections for color development. After the color development was stable, the sections were rinsed with PBS. The sections were successively stained with hematoxylin, treated with the differentiation solution, dehydrated with ethanol, and clarified with xylene. Finally, they were mounted with neutral gum and observed under a microscope.

### Western blotting

The culture medium was removed, and the cells were washed three times with pre-cooled PBS. After the cells were digested with trypsin and collected, an appropriate amount of cell lysis buffer and PMSF protease inhibitor were added. The mixture was homogenized and gently shaken at 4 °C for 45 min. The supernatant was obtained by centrifugation, 5× protein loading buffer was added, boiled for 10 min, and then stored in a −80 °C low-temperature freezer. The tissue was minced and added to pre-cooled PBS. It was processed using a homogenizer and left to stand for 5 min. The supernatant was discarded after centrifugation. Strong RIPA lysis buffer and PMSF protease inhibitor were added, mixed well, and shaken on ice for 45 min. The supernatant was collected again after centrifugation. 5× protein loading buffer was added, boiled for 10 min, and then stored in a −80 °C low-temperature freezer. The samples were added, and electrophoresis was performed. The gel was cut and transferred to a membrane. The PVDF membrane was first depolarized, followed by rehydration. Subsequently, it was blocked and incubated with primary and secondary antibodies. Lastly, development and detection procedures were performed.

### Real-time quantitative PCR (RT-PCR)

After cells were digested with trypsin, the cell suspension was transferred to RNase-Free EP tubes. The cell pellet was collected by centrifugation and then lysed by adding Trizol. After being left to stand at room temperature for 5 min, chloroform was added and shaken vigorously. The aqueous phase was separated by centrifugation at 4 °C. The aqueous phase was transferred to a new tube, and isopropanol was added to precipitate RNA. After being left to stand at room temperature, the RNA was centrifuged and washed twice. The RNA precipitate was dried, dissolved in RNase-free water, and incubated at 55–60 °C. Finally, it was stored at −80 °C for future use. A small amount of tissue pieces were placed in an RNase-Free EP tube and washed twice with PBS. The tissue was minced using sterilized scissors, and 1 mL of Trizol reagent was added. The sample was processed using a homogenizer. The subsequent steps were in accordance with the method for extracting total RNA from cells. THiScript^®^ II reverse transcriptase was employed to synthesize cDNA. The cDNA product was diluted fivefold. The PCR mixture was centrifuged at 3500 r/min for 3 min in a centrifuge and placed in a PCR instrument for analysis using the 2^−△△CT^ method.

### RNA-binding protein immunoprecipitation (RIP)

Pre-cooled PBS was employed to resuspend the cells twice to remove the supernatant, and finally, 1 mL of PBS was used for resuspension again. 37% formaldehyde was added to the cell solution until the final concentration reached 1%, and the mixture was incubated with slow rotation at room temperature for 10 min. Subsequently, glycine solution was added to achieve a final concentration of 0.25 M to quench the cross-linking reaction, and the incubation was continued at room temperature for 5 min. The cells were washed twice with pre-cooled PBS, and 1 mL of the prepared RIPA lysis buffer was added for resuspension. The cells were lysed by three rounds of sonication. 50 μL was taken as input, added to Trizol, and stored at −80 °C. The protein and mRNA complex in lysis was precleaned by protein A. Protein A and associated primary antibodies were washed with RIPA buffer before immunoprecipitation. The immunoprecipitation process was mixed by rotation at 4 °C overnight. The mixture was centrifuged at low speed for 5 min, and the supernatant was discarded. The beads containing the immunoprecipitated samples were collected and resuspended in 100 μL of the decrosslinking agent, and incubated in a 70 °C water bath for 45 min to reverse the cross-linking. Trizol was added to the solution, and RNA was extracted.

### Transcript sequencing

The total RNA was extracted from the constructed stable cell lines and sent to the company (Shanghai Ouyi Biomedical Technology Co., Ltd.) for RNA sequencing to generate an RNA-seq dataset. Quality control and filtering were performed on the sequencing data to eliminate low-quality reads and contaminated sequences. The target genes were analyzed using the GSEA program (http://www.gsea-msigdb.org/gsea/login.jsp).

### Immunofluorescence staining

After the tissue samples were fixed, embedded, and sectioned, 500 μL of 1% BSA was added to each section and incubated at room temperature for 2 h for blocking. The blocking solution was removed, and 200 μL of primary antibody was added and incubated at 4 °C overnight. After the removal of the primary antibody, the sections were washed three times with PBS. 200 μL of fluorescent secondary antibody was added and incubated at room temperature for 2 h. After the removal of the secondary antibody, the sections were washed three times again with PBS. 200 μL of Hochest was added and incubated at room temperature for 10 min, followed by three washes with PBS. Anti-fading agent was dropped, a coverslip was covered, and the sections were dried in the dark for 5 min, then they could be observed under a fluorescence microscope.

### Multiplex immunofluorescence

Following fixation, embedding, and sectioning of the tissue samples, 500 μL of 1% BSA was applied to each section and incubated at room temperature for 2 h to block non-specific binding sites. The blocking solution was subsequently discarded. The specific primary antibody was then diluted using the designated primary antibody diluent, and 200 μL of this solution was added to each section, followed by incubation at room temperature for 1 h. After removal of the primary antibody, the sections were washed three times with PBS. Next, 200 μL of the designated fluorescent secondary antibody was added, and the sections were incubated at room temperature for 10 min. The secondary antibody was then removed, and the sections were washed three additional times with PBS. This process was repeated to sequentially stain multiple protein molecules. Finally, an anti-fading mounting medium containing DAPI was applied, the coverslip was placed, and the preparation was allowed to dry in the dark for 10 min before observation under a fluorescence microscope.

### Flow cytometry

Cells were collected in 2 mL EP tubes, washed twice with PBS, and resuspended in 100 μL FACS buffer. Subsequently, 0.5 μL Fc-block was added, and the cells were incubated on ice for 10 min. Next, 0.5 μL of the surface molecule antibody was added, and the cells were incubated on ice in the dark for 30 min. The cells were then washed twice with FACS buffer and resuspended in 200 μL IC fixation buffer, followed by incubation at room temperature in the dark for 30 min. The cells were washed twice with permeabilization buffer, resuspended in 100 μL permeabilization buffer, and 0.5 μL of the intracellular staining antibody was added. The cells were incubated on ice in the dark for 30 min. Finally, the cells were washed twice with permeabilization buffer, resuspended in 200 μL PBS, and analyzed using a flow cytometer.

### Preprocessing, integration, and clustering of single-cell RNA-seq data

Gene-cell matrixes were filtered to remove cells: (1) The number of detected genes per cell was required to be greater than 300 and less than 7000; (2) The UMI count per cell was required to be greater than 1000, with the top 3% of cells being excluded based on their total counts; (3) The percentage of mitochondrial gene expression relative to the total gene expression per cell was required to be less than 10%; (4) The percentage of hemoglobin gene expression relative to the total gene expression per cell was required to be less than 3%. Then the matrix was imported into the R package Seurat (v 5.10) for subsequent analysis.

The merged single-cell RNA-seq dataset was normalized using the Normalize Data function. Highly variable features were identified using the Find Variable Features function to select genes with the most variation across cells. To eliminate the potential effects of mitochondrial gene expression, the data were regressed using Scale Data while correcting for the percentage of mitochondrial gene expression (mt_percent). Principal component analysis (PCA) was then performed on the scaled data using the Run PCA function, with verbosity turned off for cleaner output. The single-cell RNA-seq data were integrated using the Integrate Layers function with the Harmony method. The principal components obtained from PCA were used as the original reduction (orig.reduction), and Harmony integration was performed to obtain a new reduction (new.reduction). To determine the appropriate number of dimensions for clustering, an Elbow Plot was generated, considering up to 50 dimensions. Neighbor graph construction was performed using the Find Neighbors function with the Harmony-reduced data (reduction = “harmony”) and the first 20 principal components (dims = 1:20). Clustering was carried out using the Find Clusters function with varying resolution values ranging from 0.1 to 1.0 to identify optimal cluster structures. Finally, UMAP dimensionality reduction was performed on the integrated Harmony-reduced data (reduction = “harmony”) using the first 20 principal components (dims = 1:20) for visualization of the clusters.

### SingleR analysis for cell Type annotation

SingleR (v 2.6.0) was then applied to assign cell type labels by comparing the gene expression profiles of the test dataset with the reference dataset (ref_Mouse). The main cell type labels from the reference were used for annotation. The SingleR function was run with clusters as the input to predict cell type labels for each cluster.

### Extraction of tumor and normal reference cells, copyKAT analysis, and visualization

Tumor-originating epithelial cells and selected normal reference cells were extracted based on SingleR annotations and merged for downstream analysis. The RNA assay was set as the default, and expression layers were joined using JoinLayers. The raw expression matrix was extracted (GetAssayData) for CopyKAT analysis. Normal reference cells (immune cells) were defined, and CopyKAT (v 1.10) was run to predict cell ploidy and infer malignancy. The prediction results were added as metadata to the Seurat object, and cell classifications were updated to classify undefined cells as “diploid.” A UMAP plot was generated (DimPlot) to visualize the CopyKAT results, and the annotated object was saved for further use.

### Epithelial cell pseudotime analysis

To analyze the differentiation trajectory of tumor cells, A Monocle object was created from a Seurat object containing tumor cells derived from epithelial cells and group 0 normal cells. The gene expression matrix (counts) and metadata were extracted, and gene annotations were prepared. These data were then converted into AnnotatedDataFrame objects, which were used to create a CellDataSet (cds) for Monocle (v 2.32.0). Size factors were estimated to normalize for differences in gene expression across cells, and gene dispersion was estimated to facilitate downstream differential expression analysis.

Lowly expressed genes were filtered out, and differential gene testing was performed to identify genes associated with cell type differentiation. Genes with a q-value < 0.01 were selected as ordering genes for pseudotime analysis. These genes were used to set the ordering filter for cell trajectory analysis. Dimensionality reduction was performed using the DDRTree method, and cells were ordered along the pseudotime trajectory. The results were visualized, and the ordering genes were plotted and saved for further examination.

### Statistical analyses

All the experiments involved in this study were repeated at least three times. All data were expressed as mean ± standard deviation, and the significant differences between the experimental group and the control group were reflected by the method of analysis of variance. GraphPad Prism 10 and SPSS 21.0 software were adopted for analysis, where * indicates *P* < 0.05; ** indicates *P* < 0.01; *** indicates *P* < 0.01.

## Supplementary information

Supplementary Figure S1

Supplementary Figure S2

Supplementary Figure S3

Supplementary Figure S4

Supplementary Figure S5

Supplementary Figure S6

Supplementary Figure S7

Supplementary Figure Legends

Original WB Images

## Data Availability

All data generated or analyzed during this study are included in this article and the supporting information files. Any additional data and original data presented in this article are available from the corresponding author upon request.
