# Supplementary figures and images for: N4BP1 is essential for the development of oral cancer via controlling both cancer cells and immune microenvironment
Source: Cell Death Dis. 2026 Jan 9;17(1):23. doi: 10.1038/s41419-025-08229-0 (PMC12789084; doi:10.1038/s41419-025-08229-0)

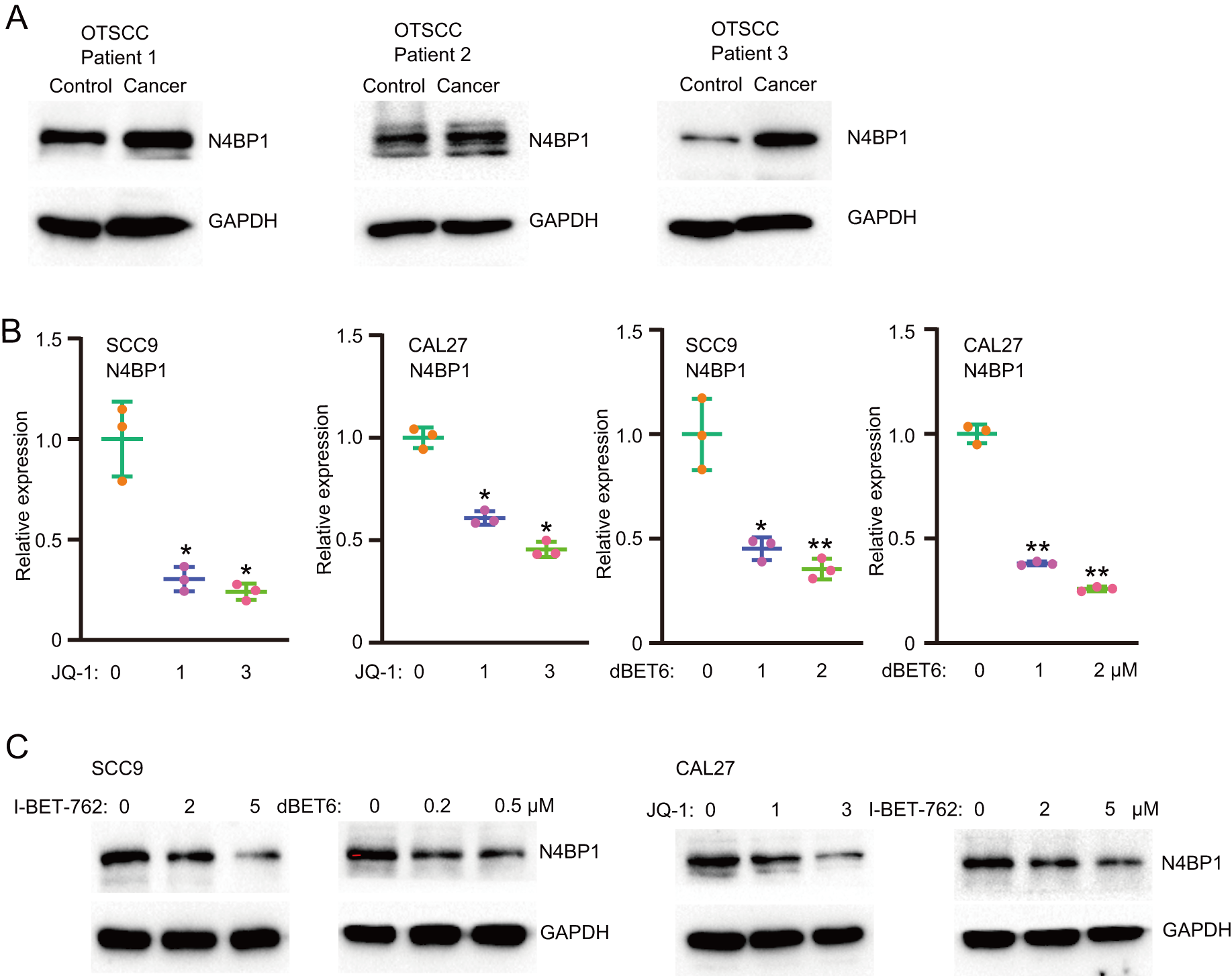

Supplement: Supplementary file 1 — Supplementary Figure S1 [file 41419_2025_8229_MOESM1_ESM.tif]

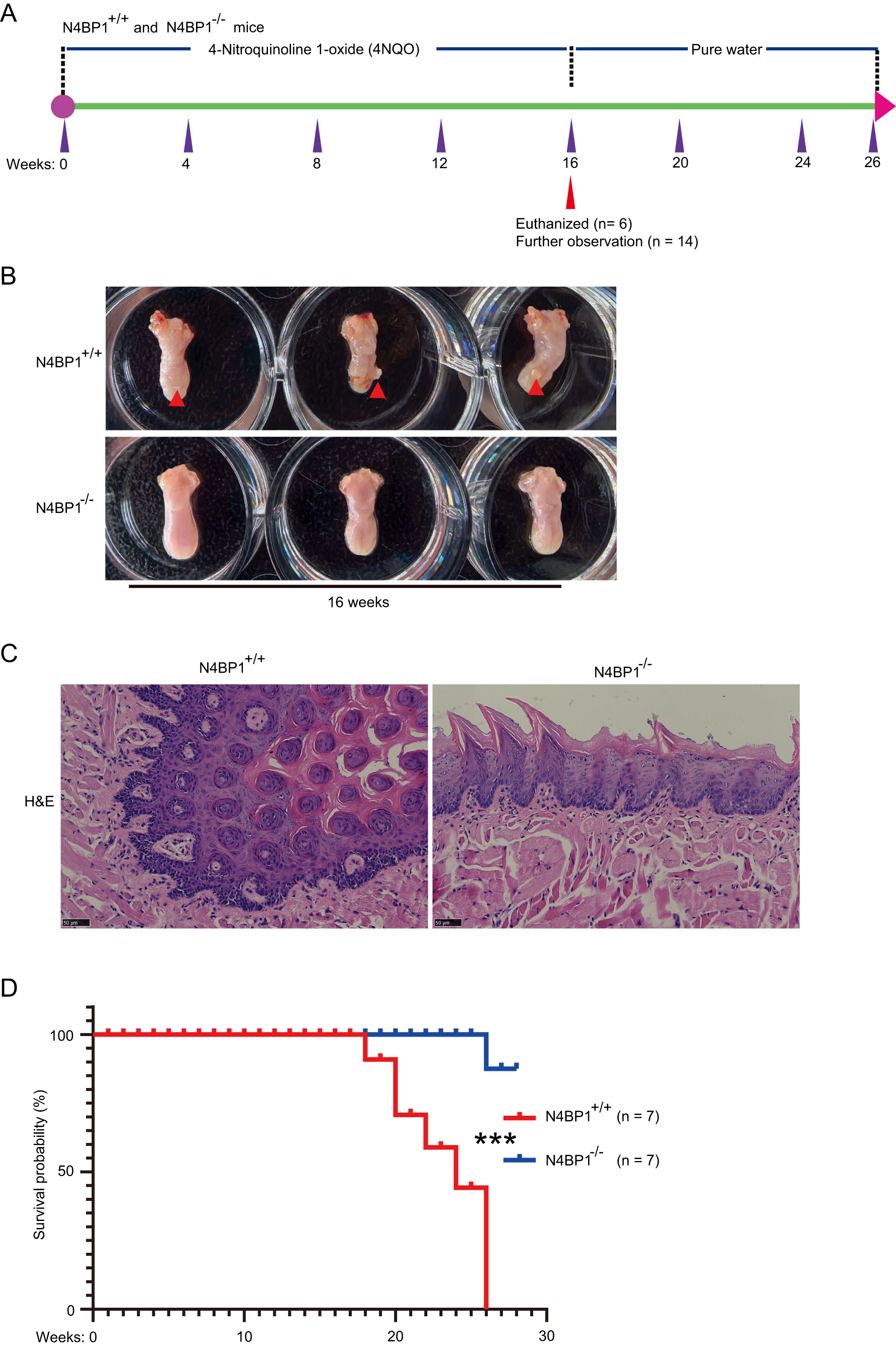

Supplement: Supplementary file 2 — Supplementary Figure S2 [file 41419_2025_8229_MOESM2_ESM.tif]

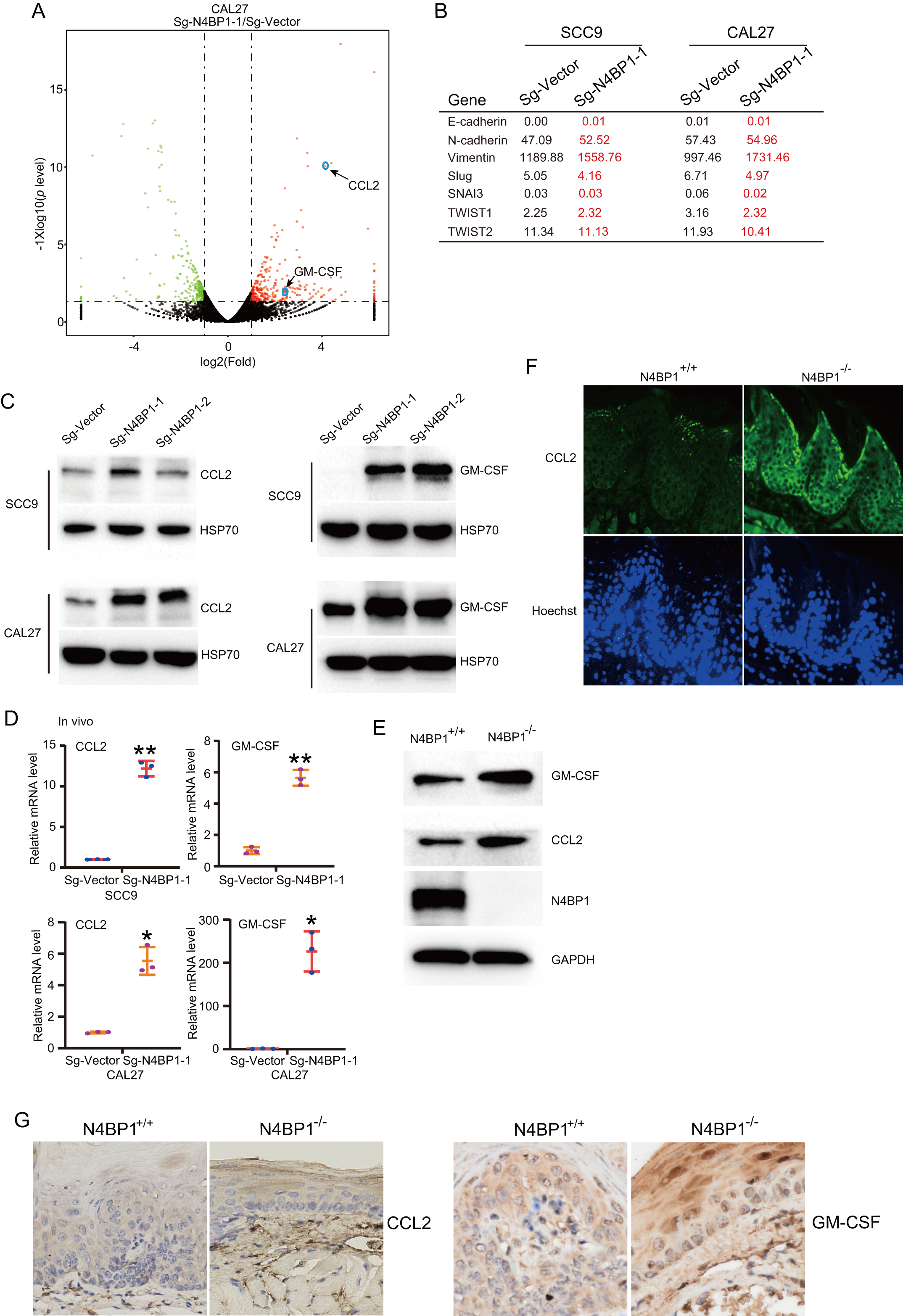

Supplement: Supplementary file 3 — Supplementary Figure S3 [file 41419_2025_8229_MOESM3_ESM.tif]

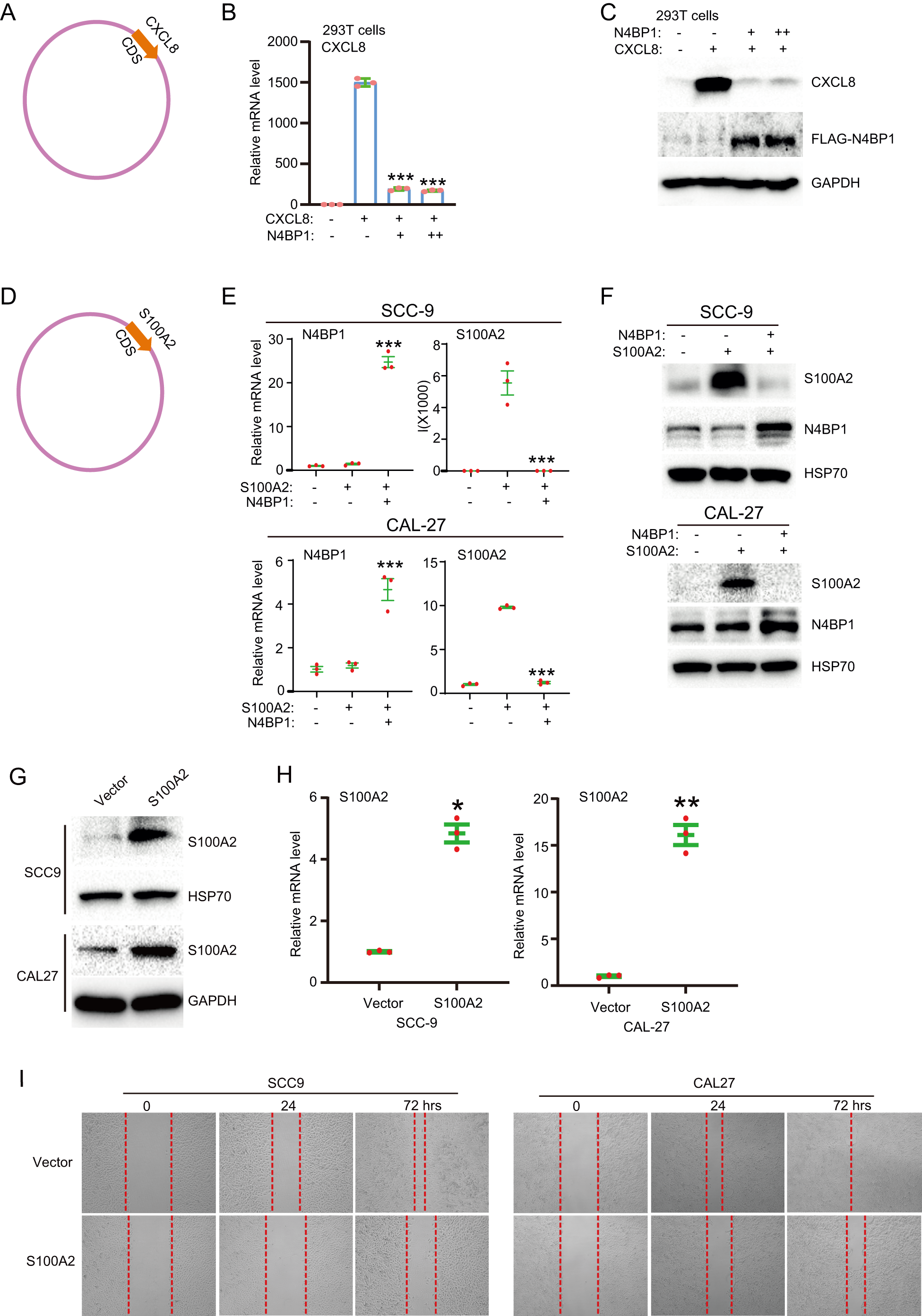

Supplement: Supplementary file 4 — Supplementary Figure S4 [file 41419_2025_8229_MOESM4_ESM.tif]

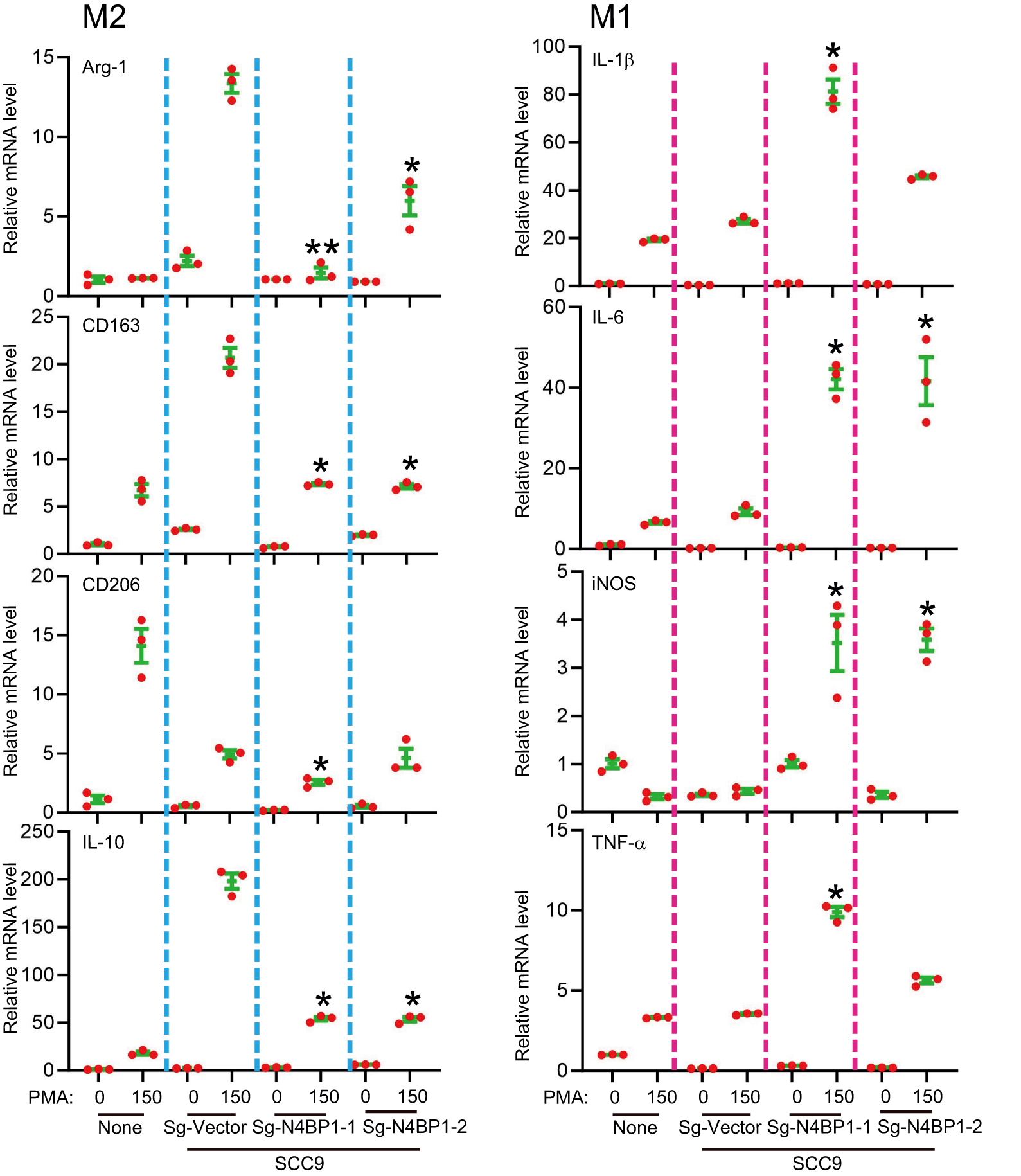

Supplement: Supplementary file 5 — Supplementary Figure S5 [file 41419_2025_8229_MOESM5_ESM.tif]

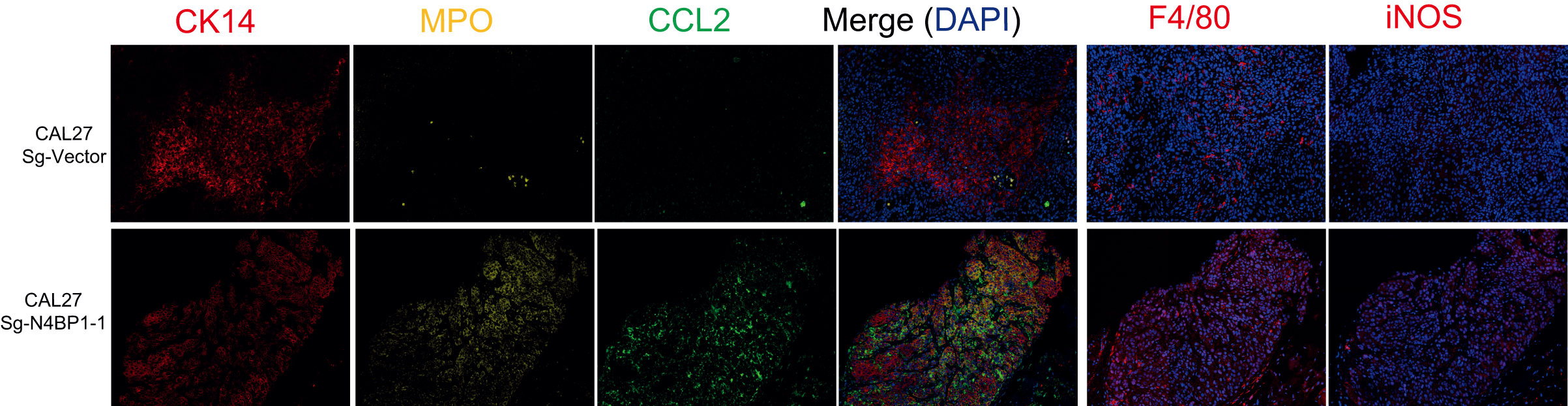

Supplement: Supplementary file 6 — Supplementary Figure S6 [file 41419_2025_8229_MOESM6_ESM.tif]

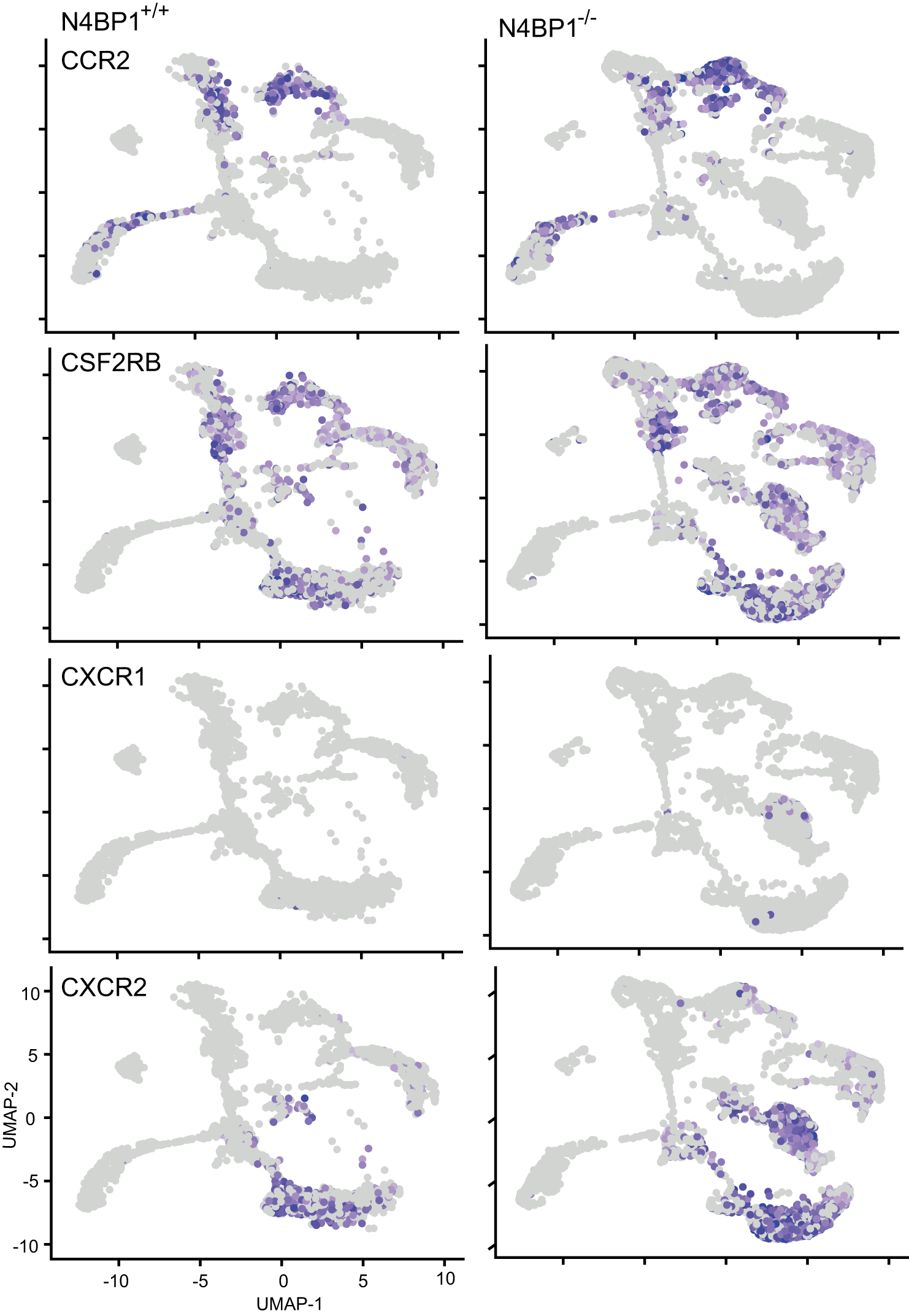

Supplement: Supplementary file 7 — Supplementary Figure S7 [file 41419_2025_8229_MOESM7_ESM.tif]
