# Supplementary material for: N4BP1 is essential for the development of oral cancer via controlling both cancer cells and immune microenvironment
Source: Cell Death Dis. 2026 Jan 9;17(1):23. doi: 10.1038/s41419-025-08229-0 (PMC12789084; doi:10.1038/s41419-025-08229-0)
Supplement: Supplementary file 8 — Supplementary Figure Legends [file 41419_2025_8229_MOESM8_ESM.docx]

**N4BP1 is essential for the development of oral cancer via controlling both cancer cells and immune microenvironment**

Yihua Song ^1,^ ^2,^ ^#^ Rong Sun ^3, #^ Jie Ji ^4, #^ Wen Zheng ^1,^ ^3^, Yanli Li ^1^, Xiaohong Guo ^1^, Liuting Chen ^1^, Yuanyuan Wu ^1^, Miaomiao Chen ^3^, Xingmei Feng ^2^, Mingbing Xiao ^4, *^, Renfang Mao ^5, *^, Yihui Fan ^1, 3, 6, *^

**Supplementary Figure Legends**

**Supplemental Figure S1**

(A) The total protein from three pairs of fresh tongue squamous cancer tissue and its non-neoplastic adjacent tissues were extracted and the protein level of N4BP1 was examined by WB; (B) SCC9 and CAL27 cells were treated with BRD4 inhibitors JQ-1 and dBET6 with indicated doses. The mRNA level of N4BP1 was determined by RT-PCR; (C) SCC9 and CAL27 cells were treated with BRD4 inhibitors JQ-1, dBET6 and I-BET-762 with indicated doses. The protein level of N4BP1 was determined by WB. Data in (B and C) are representative of three independent experiments. * p < 0.05; * * p < 0.01.

**Supplemental Figure S2**

Schematic presentation of 4-NQO-induced mice oral cancer model. Wild-type and N4BP1-deficient C57BL/6 mice were administered 4-NQO in drinking water continuously for 16 weeks, followed by a recovery period with pure water, and their survival was monitored; (B) Representative photos of 4-NQO treated tongues at week 16 from wild-type and N4BP1-deficient mice; (C) H&E staining of 4-NQO-treated wild-type and N4BP1-deficient tongue tissues; (D) Survival curve of 4-NQO-treated wild-type and N4BP1 deficient mice with 28 weeks period (n=7). Data in (B and C) are representative of four independent experiments. * * * p < 0.001.

**Supplemental Figure S3**

(A) The profiling of gene expression was performed by RNA-sequencing in CAL27 cells. The volcano plot was used to present the results and the expression of CCL2 and GM-CSF was labeled by arrows; (B) The markers of EMT-related genes in N4BP1 deficient SCC9 and CAL27 cells were listed; (C) The protein level of CCL2 and GM-CSF was determined by WB in N4BP1 wild-type and knockout SCC9 and CAL27 cells; (D) The mRNA level of CCL2 and GM-CSF in subcutaneous tumor from N4BP1 wild-type and knockout SCC9 and CAL27 cells was determined by RT-PCR; (E) The protein level of CCL2 and GM-CSF in 4-NQO-treated wild-type and N4BP1-deficient tongue tissues was determined by WB; (F) The protein level of CCL2 in 4-NQO-treated wild-type and N4BP1-deficient tongue tissues was determined by IF; (G) The protein level of CCL2 and GM-CSF in 4-NQO-treated wild-type and N4BP1-deficient tongue tissues was determined by IHC. Data in (C-G) are representative of three independent experiments. * p < 0.05; * * p < 0.01.

**Supplemental Figure S4**

Schematic diagram to show the plasmid encoding coding sequences of CXCL8; (B) 293T cells were transfected with plasmids containing CDS from CXCL8 with different amount of plasmids encoding N4BP1. The mRNA level was determined by RT-PCR; (C) 293T cells were transfected with plasmids containing CDS CXCL8 with different amount of plasmids encoding N4BP1. The protein level was determined by WB; (D) Schematic diagram to show the plasmid encoding coding sequences of S100A2; (E) SCC9 and CAL27 cells were transfected with plasmids containing CDS from S100A2 with plasmids encoding N4BP1. The mRNA level was determined by RT-PCR; (F) SCC9 and CAL27 cells were transfected with plasmids containing CDS S100A2 with plasmids encoding N4BP1. The protein level was determined by WB; (G) Plasmids encoding S100A2 was transfected in SCC9 and CAL27 cells. The stable cell lines was established by puromycin selection. The protein level was determined by WB; (H) The mRNA level from stable cell lines was examined by RT-PCR; (I) Ectopic expressed S100A2 cells were subjected to Scratch-healing assay. Data in (B、C、E and F) are representative of three independent experiments. * p < 0.05; * * p < 0.01; * * * p < 0.001.

**Supplemental Figure S5**

THP-1 cells were activated by PMA before co-cultured with control and N4BP1-deficient SCC9 and CAL27 cells. The M2 markers including Arg-1, CD163, CD206 and IL-10 was examined by RT-PCR. The M1 markers including IL-1β, IL-6, iNOS and TNF-α was examined by RT-PCR. Data are representative of three independent experiments. * p < 0.05; * * p < 0.01.

**Supplemental Figure S6**

Multiplex immunohistochemistry was performed in subcutaneous tumors from control and N4BP1-deficient SCC9 and CAL27 cells; The markers including CK14, MPO, CCL2, F4/80 and iNOS were examined.

**Supplemental Figure S7**

The mRNA level of CCR2, CSF2RB, CXCR1 and CXCR2 from single cell sequencing was analyzed. Their expression in different cell populations was shown.
