# Supplementary material for: N4BP1 is essential for the development of oral cancer via controlling both cancer cells and immune microenvironment
Source: Cell Death Dis. 2026 Jan 9;17(1):23. doi: 10.1038/s41419-025-08229-0 (PMC12789084; doi:10.1038/s41419-025-08229-0)

Original WB image

Fig. 1F

N4BP1


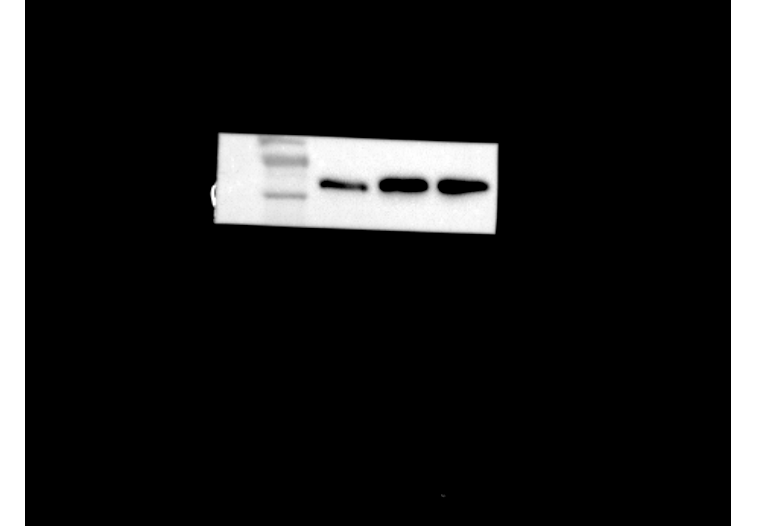


GAPDH


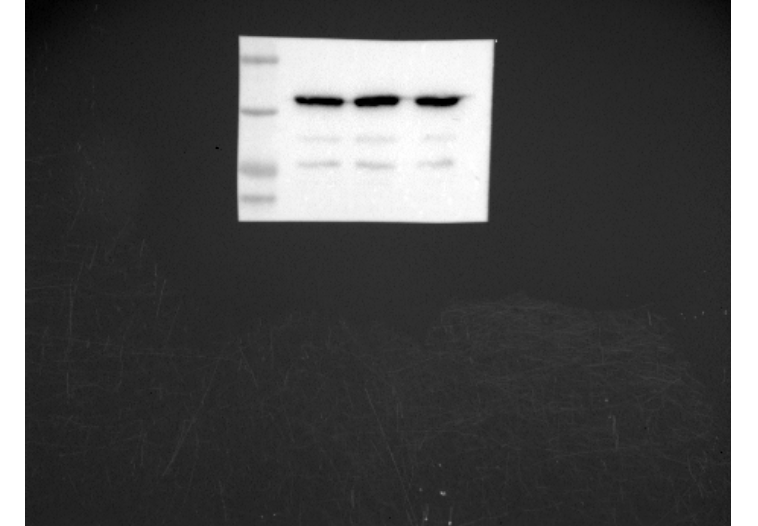


Fig 3A

SCC9-N4BP1


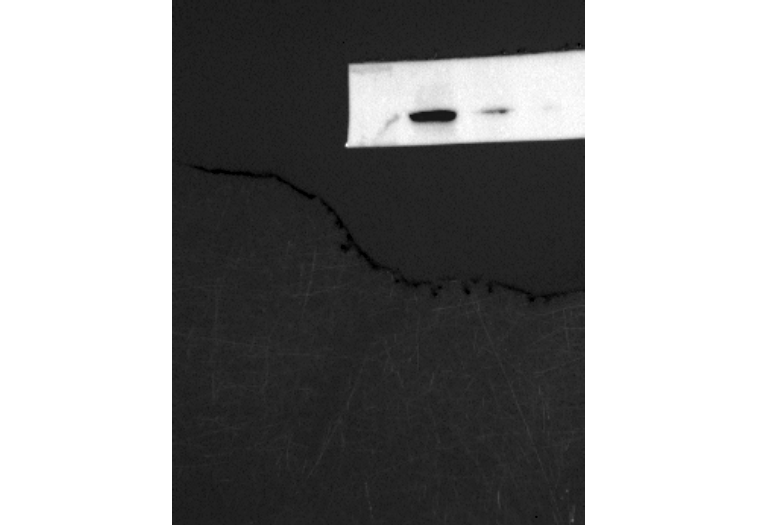


SCC9-GAPDH


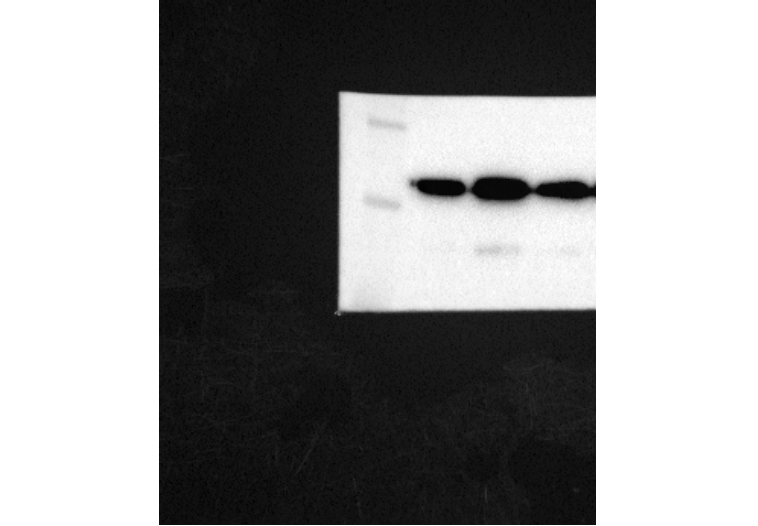


CAL27-N4BP1


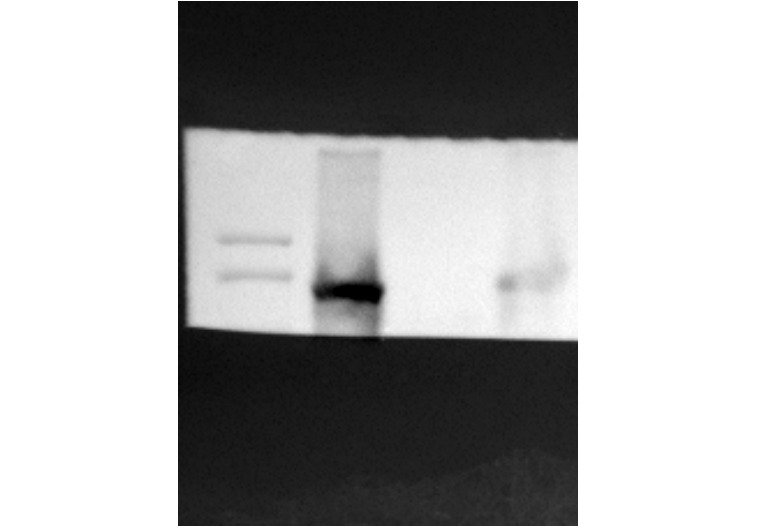


CAL27-GAPDH


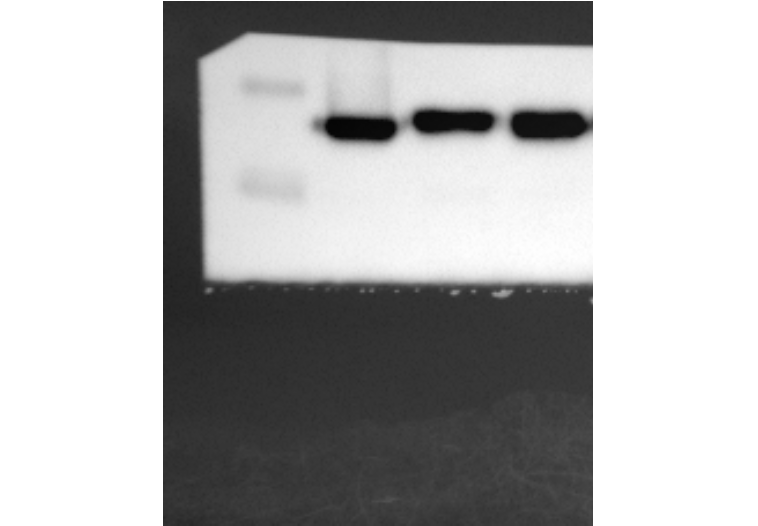


Fig 3E

SCC9-N4BP1


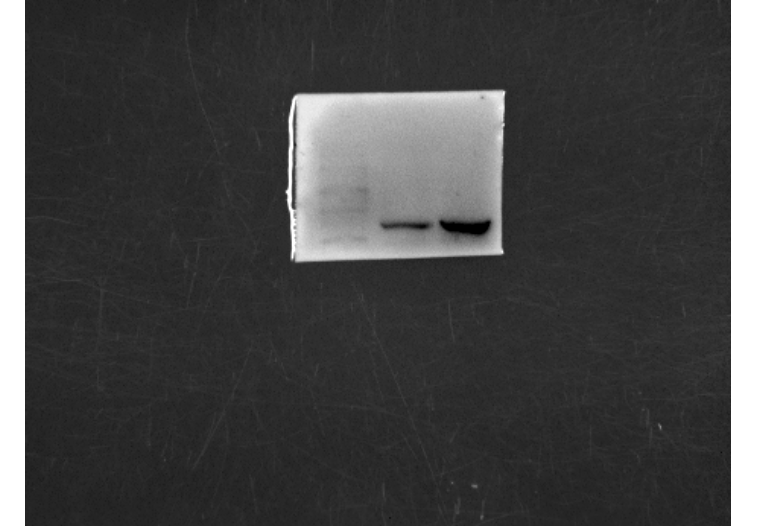


SCC9-GAPDH
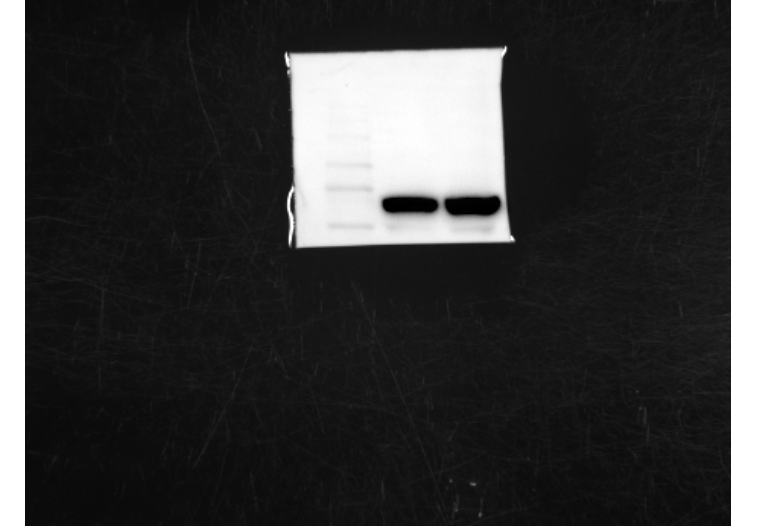


CAL27-N4BP1


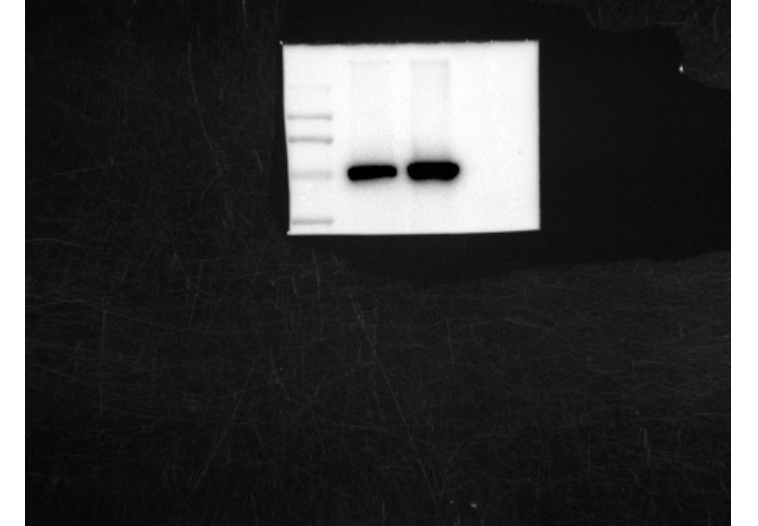


CAL27-GAPDH


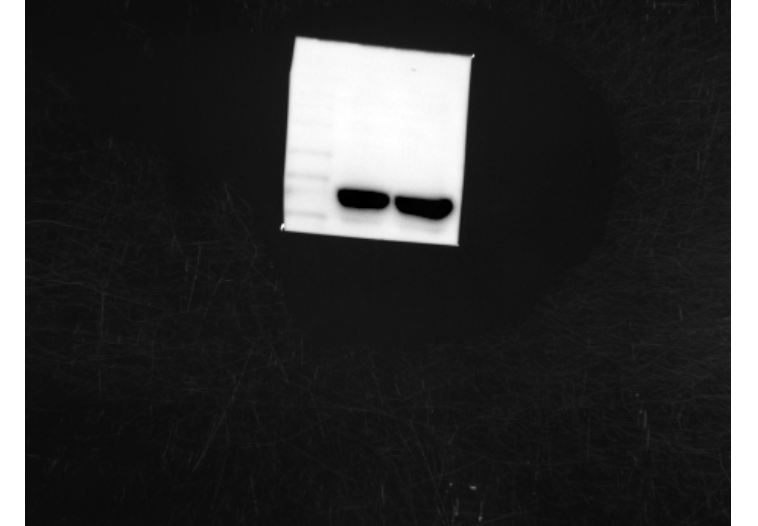


Fig 5D

SCC9-CCL2


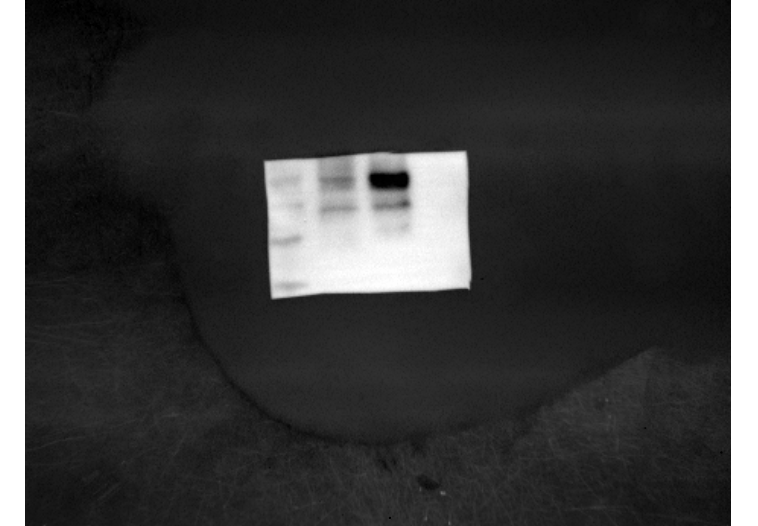


SCC9-GAPDH


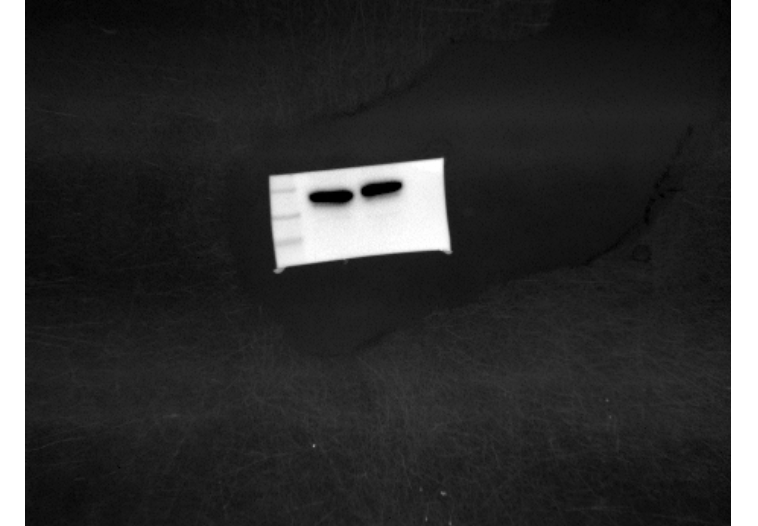


CAL27-CCL2


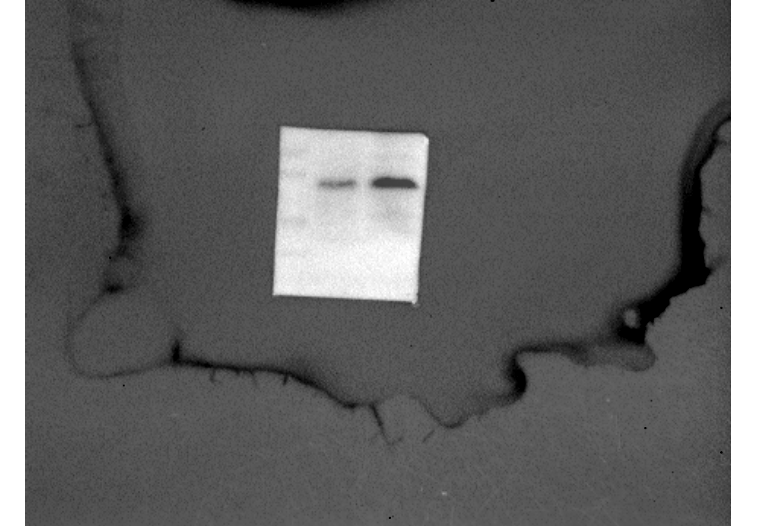


CAL27-GAPDH


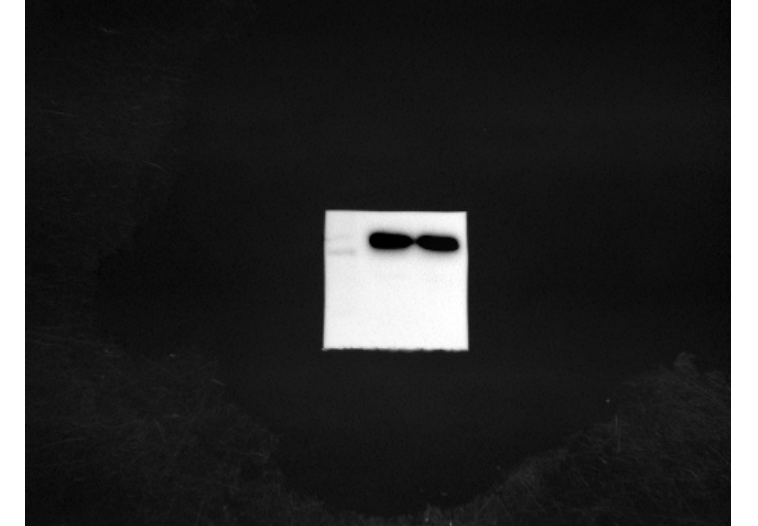


SCC9-GM-CSF


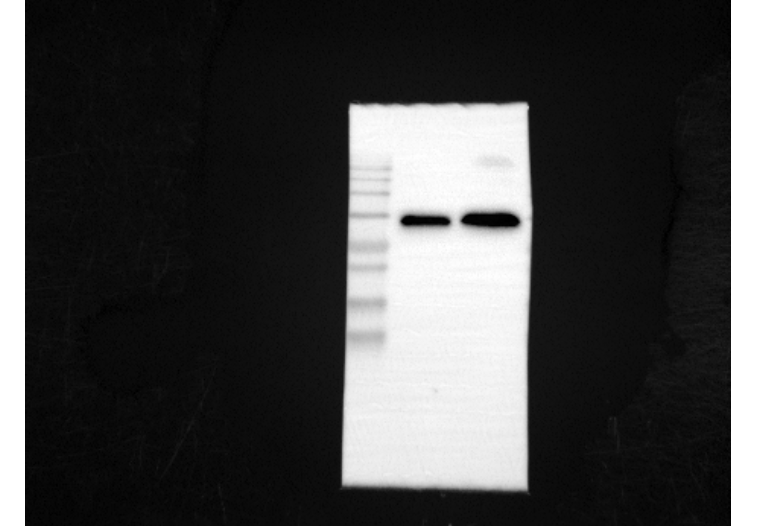


SCC9-HSP70


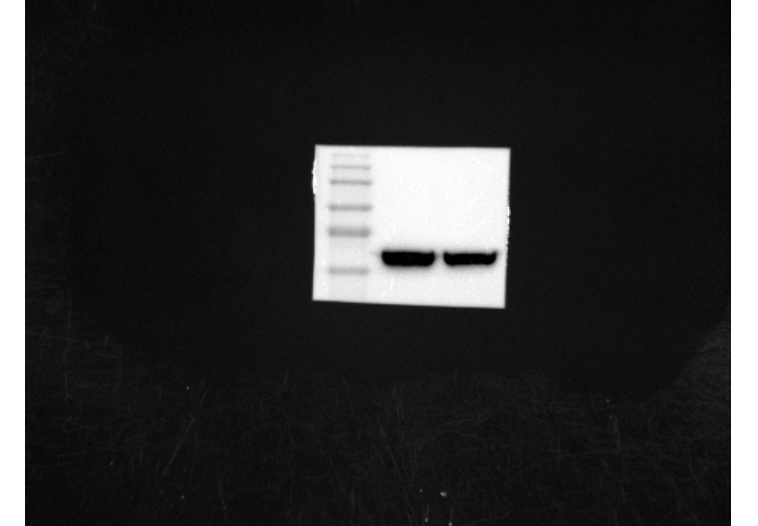


CAL27-GM-CSF


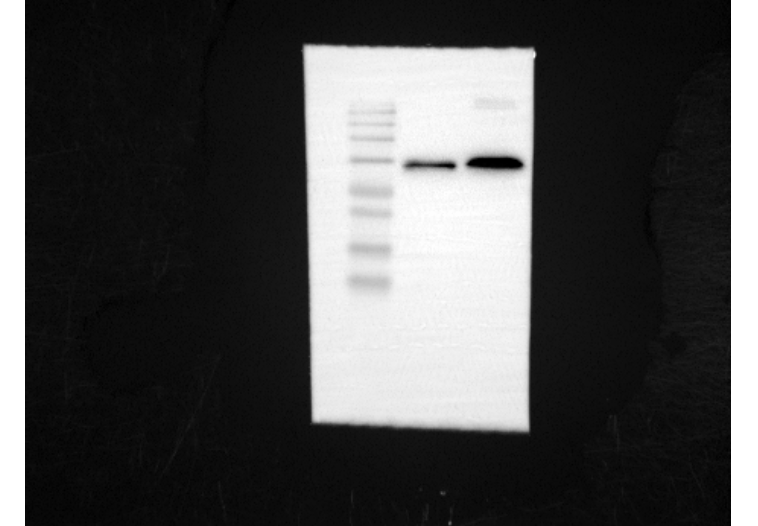


CAL27-HSP70


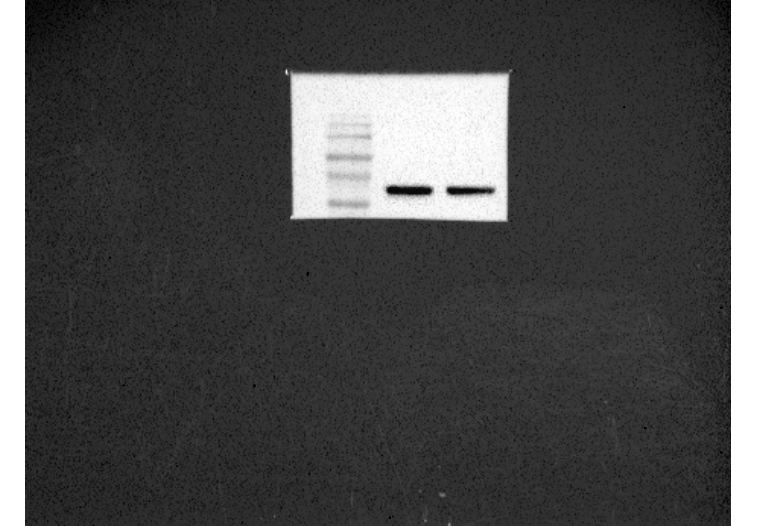


Fig 6C

CCL2


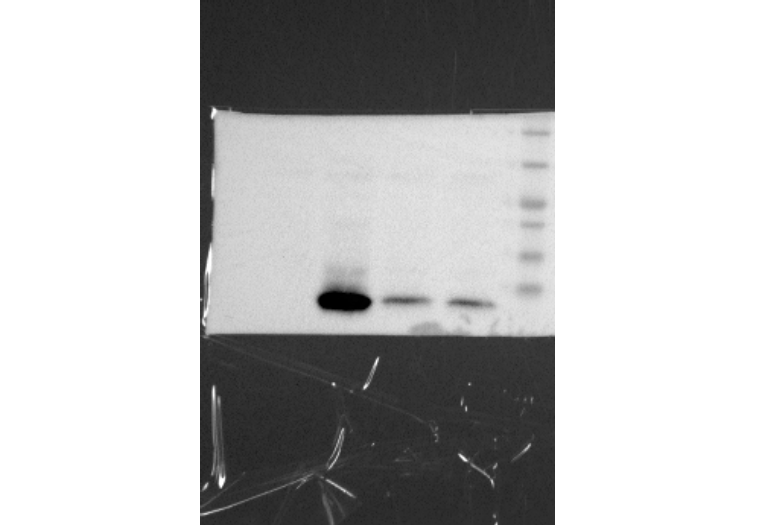


GM-CSF


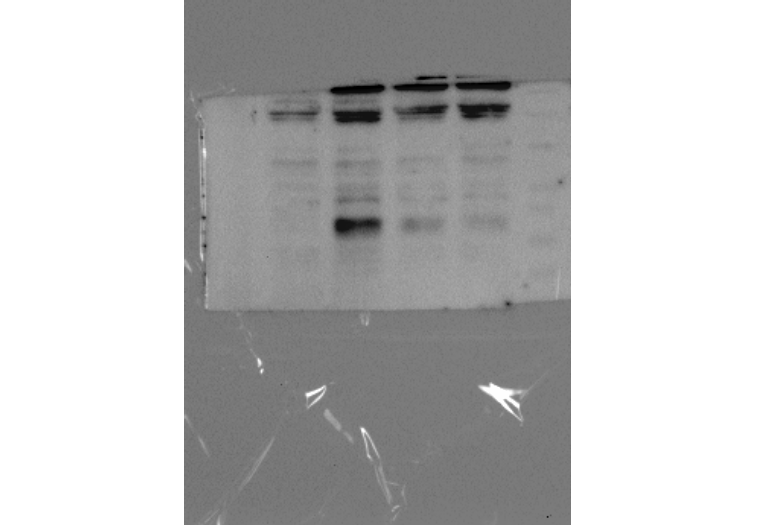


FLAG-N4BP1


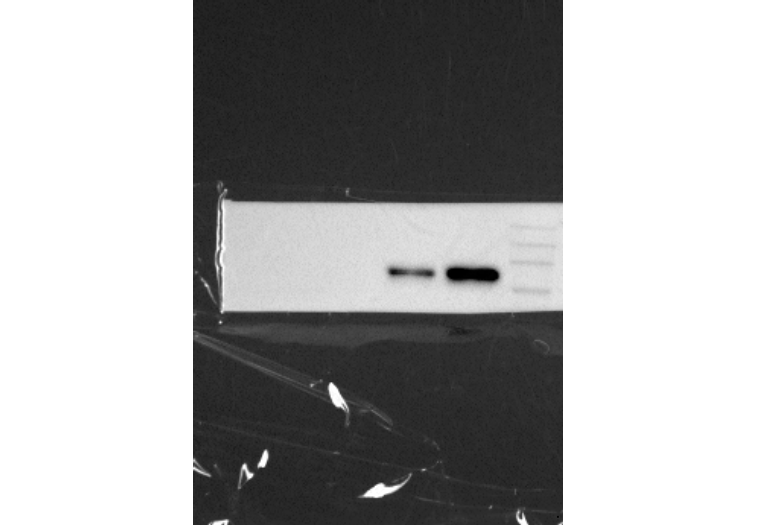


HSP70


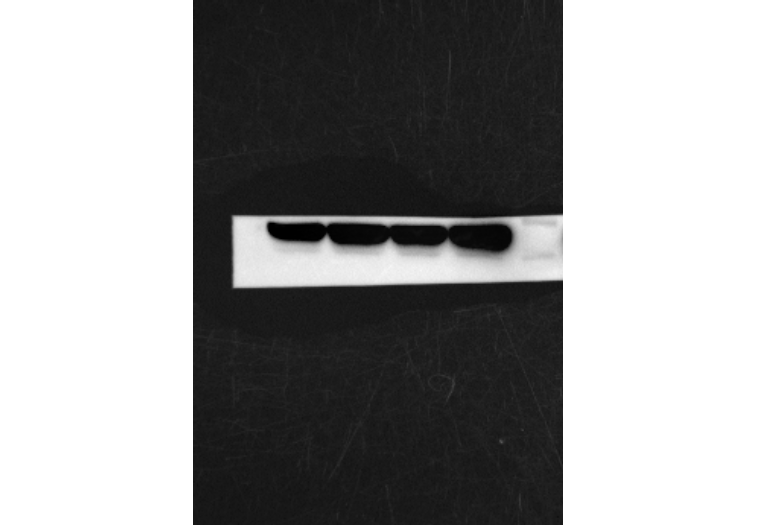


Fig S1A

Patient 1-N4BP1


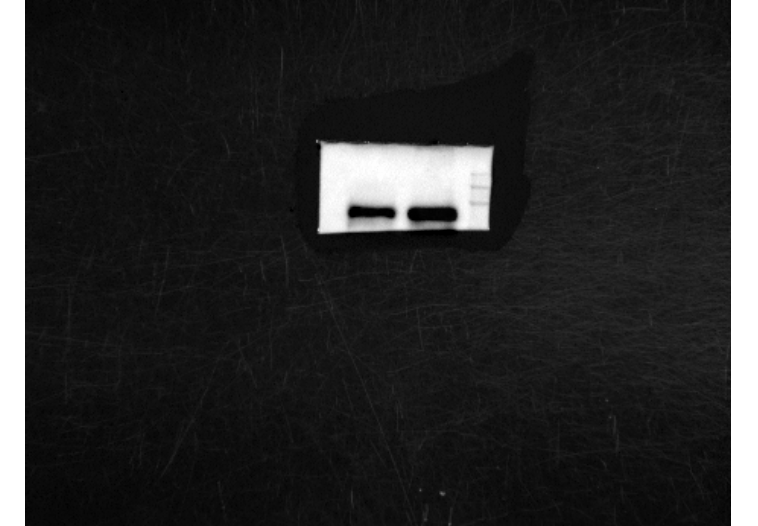


Patient 1-GAPDH


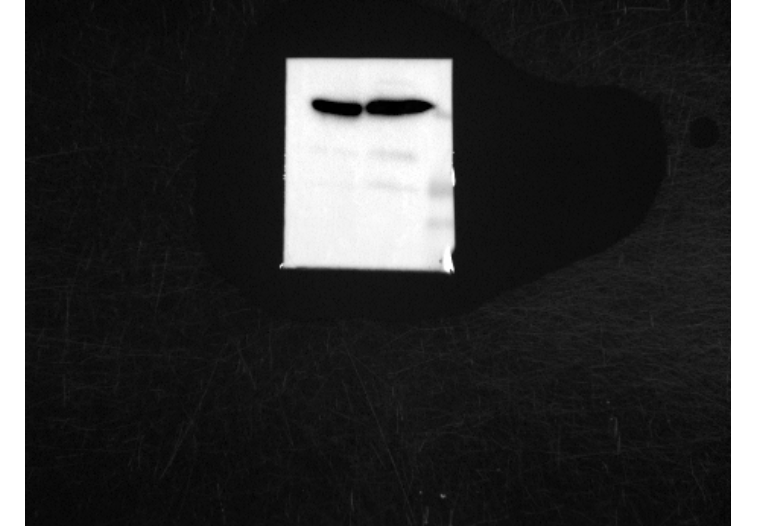


Patient 2-N4BP1


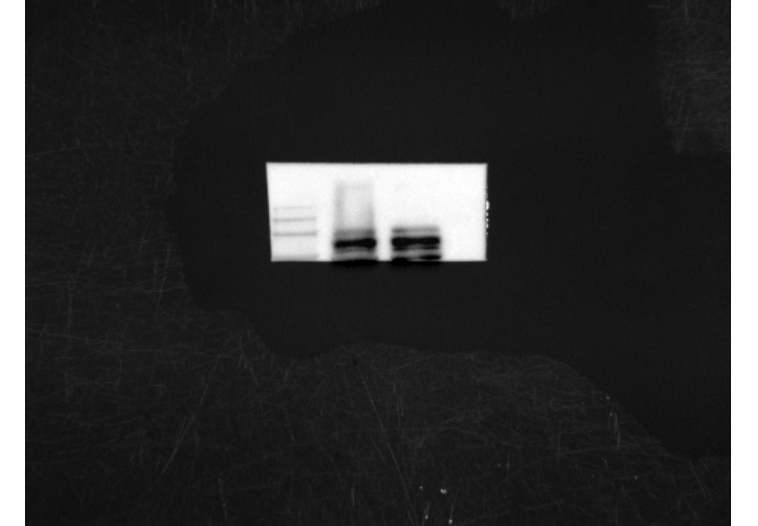


Patient 2-GAPDH


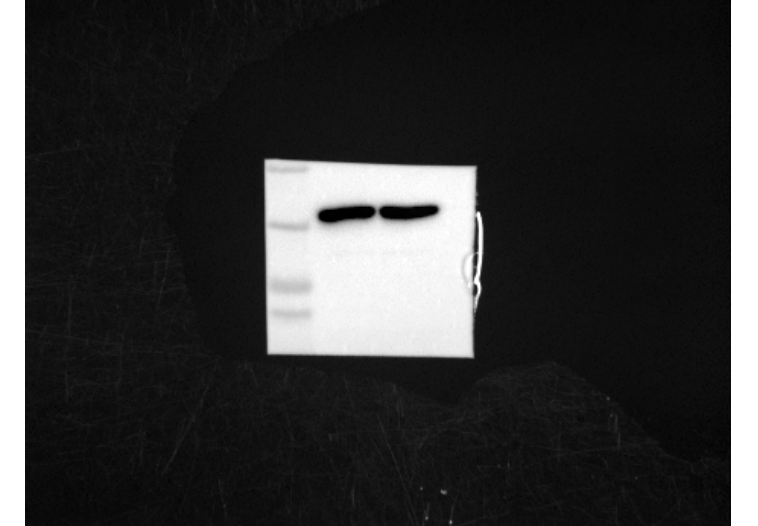


Patient 3-N4BP1


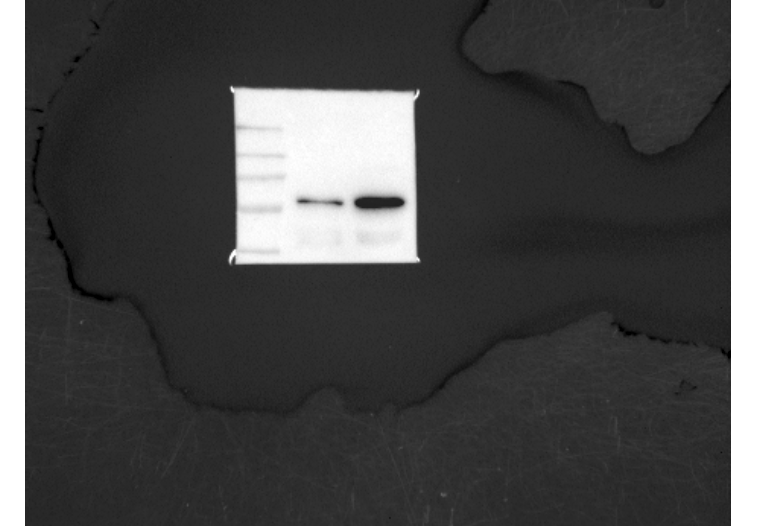


Patient 3-GAPDH


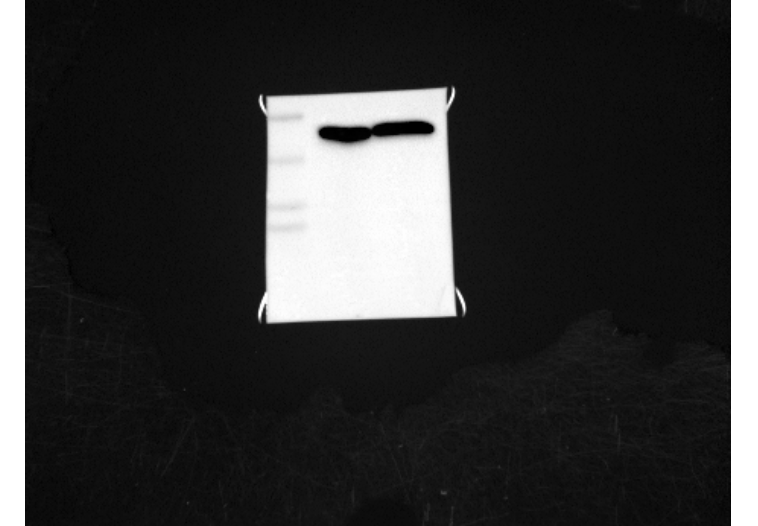


Fig S1C

SCC9-I-BET-762-N4BP1


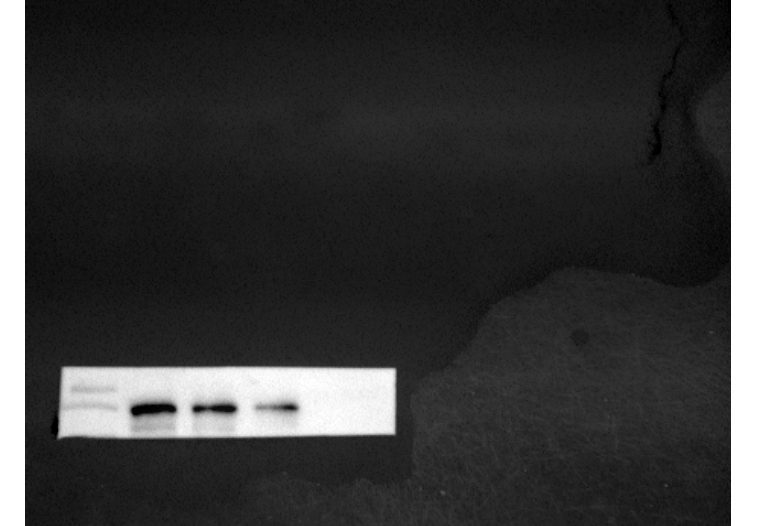


SCC9-I-BET-762-GAPDH


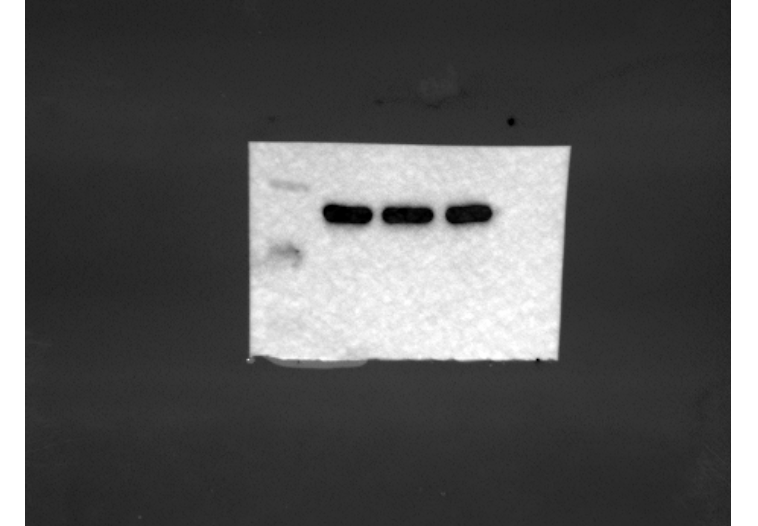


SCC9-dBET6-N4BP1


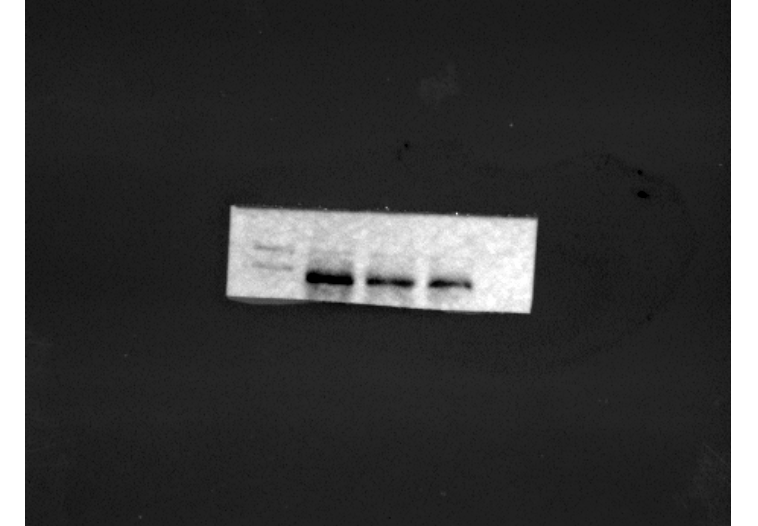


SCC9-dBET6-GAPDH


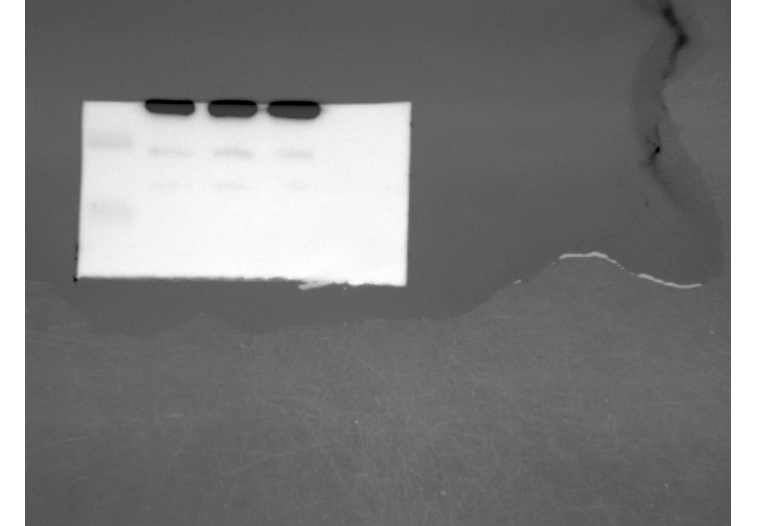


CAL27-JQ1-N4BP1


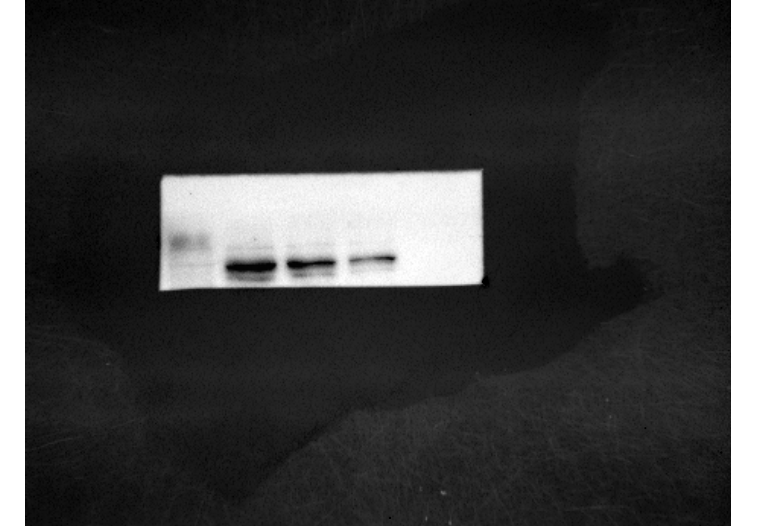


CAL27-JQ1-GAPDH


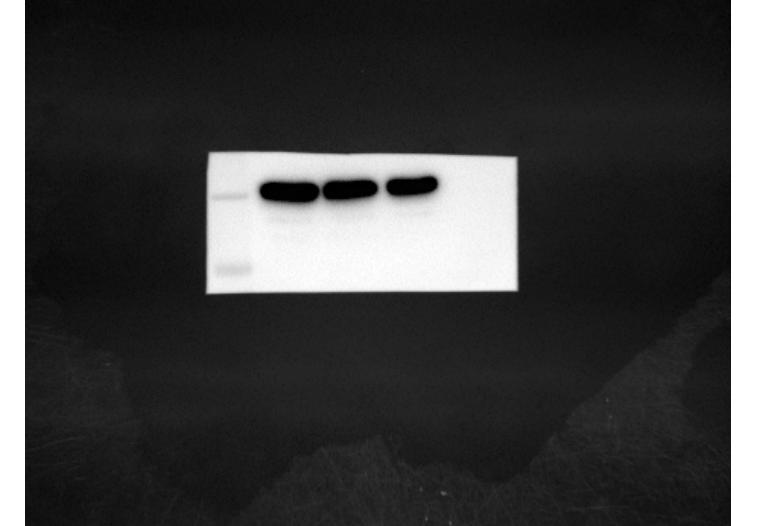


CAL27-I-BET-762-N4BP1


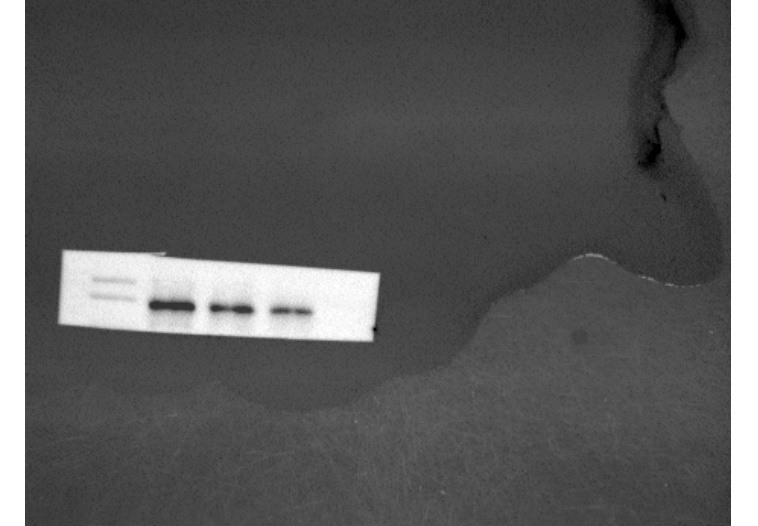


CAL27-I-BET-762-GAPDH


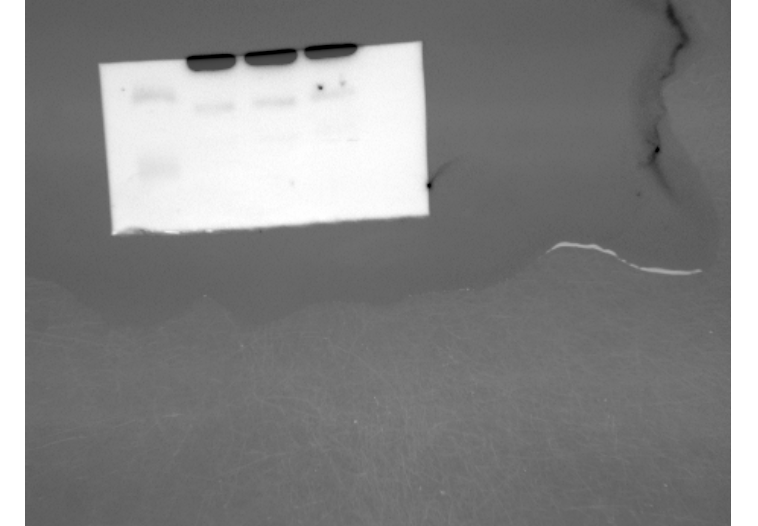


Fig S3C

SCC9-CCL2


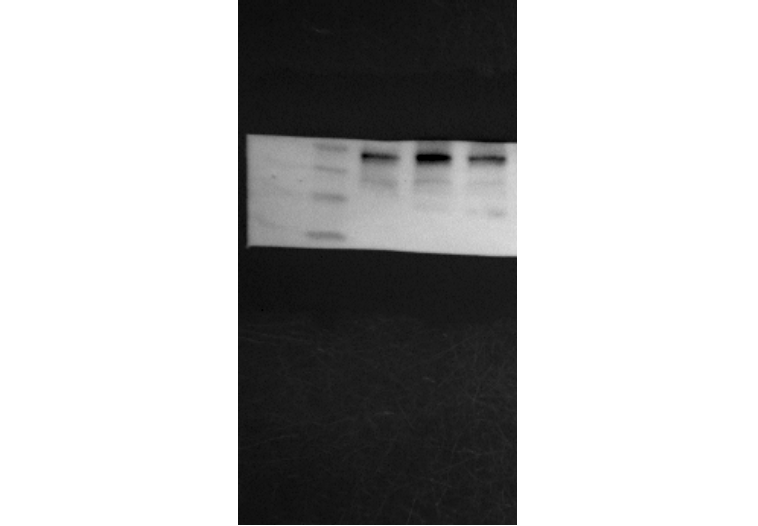


SCC9-HSP70


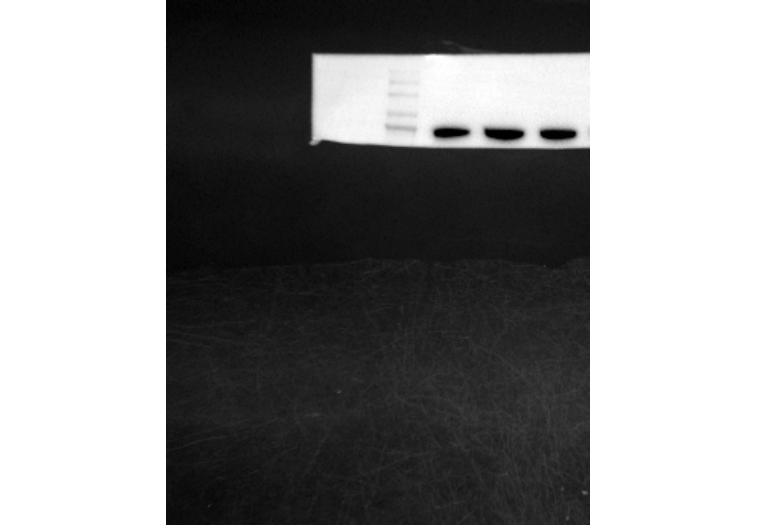


CAL27-CCL2


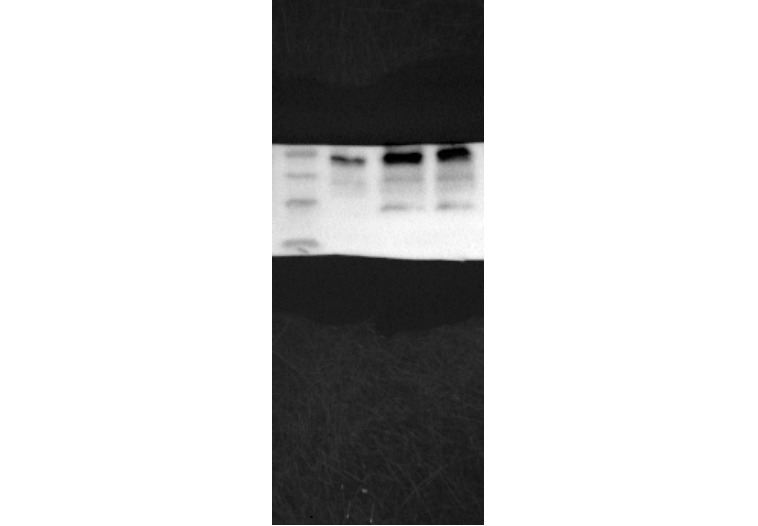


CAL27-HSP70


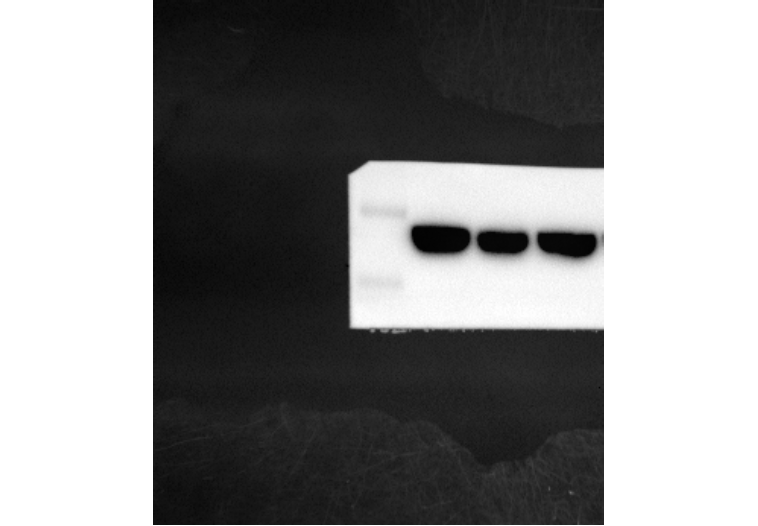


SCC9-GM-CSF


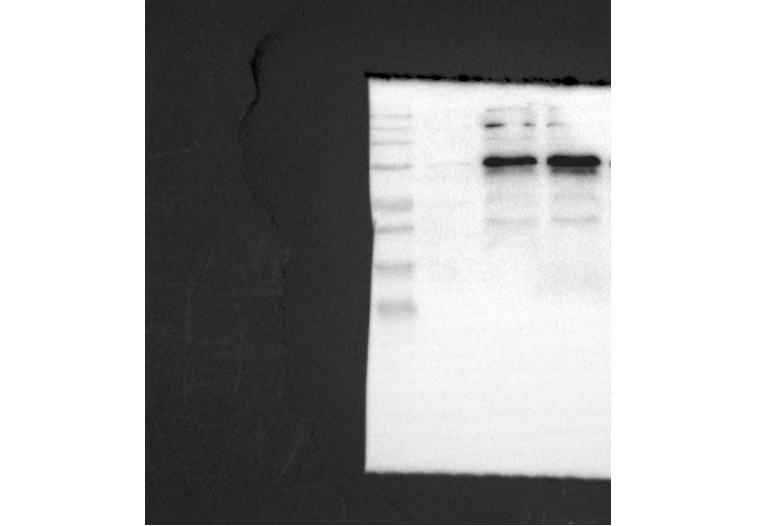


SCC9-HSP70


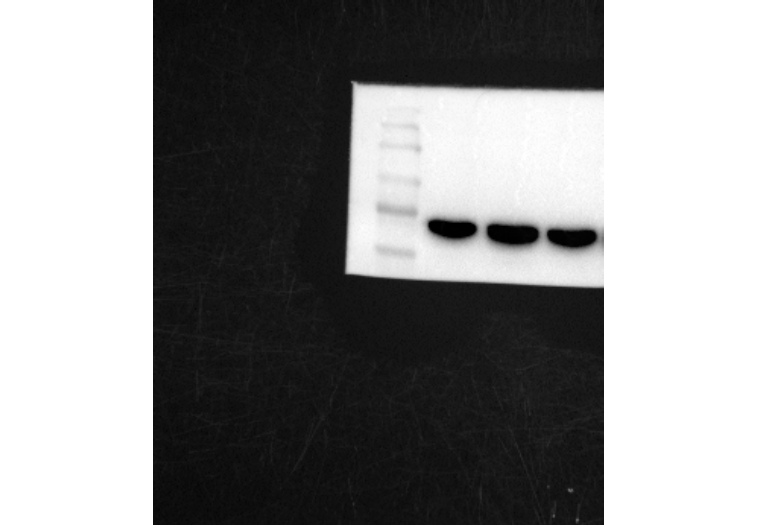


CAL27-GM-CSF


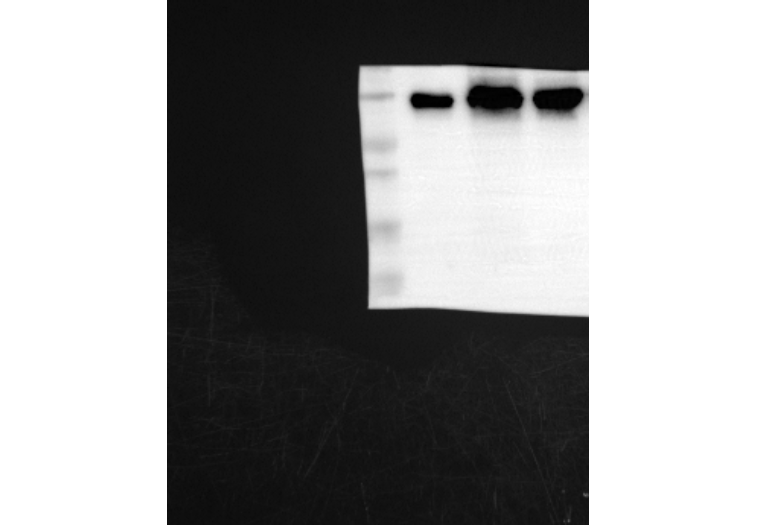


CAL27-HSP70


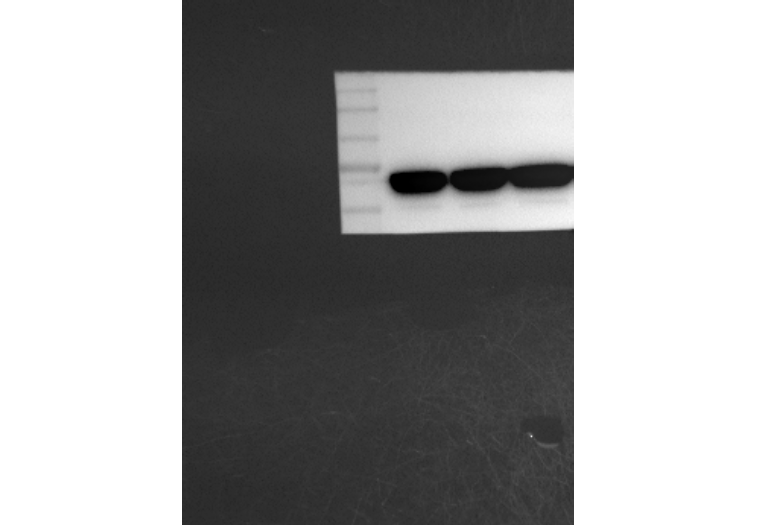


Fig S3E

GM-CSF


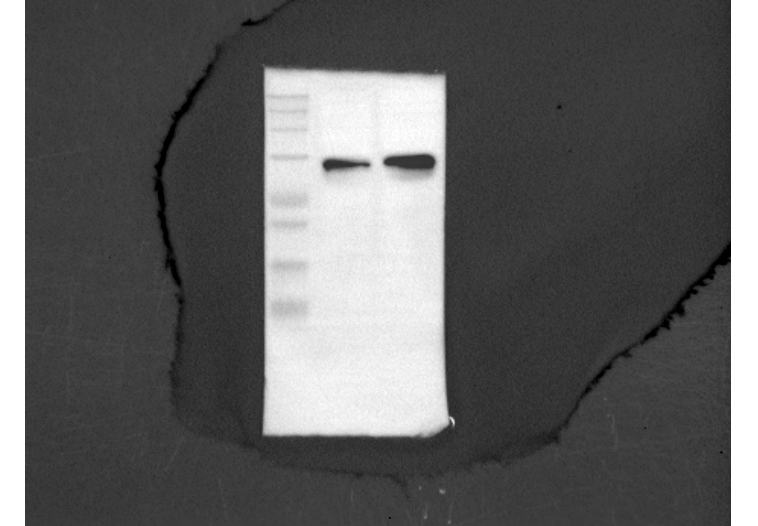


CCL2


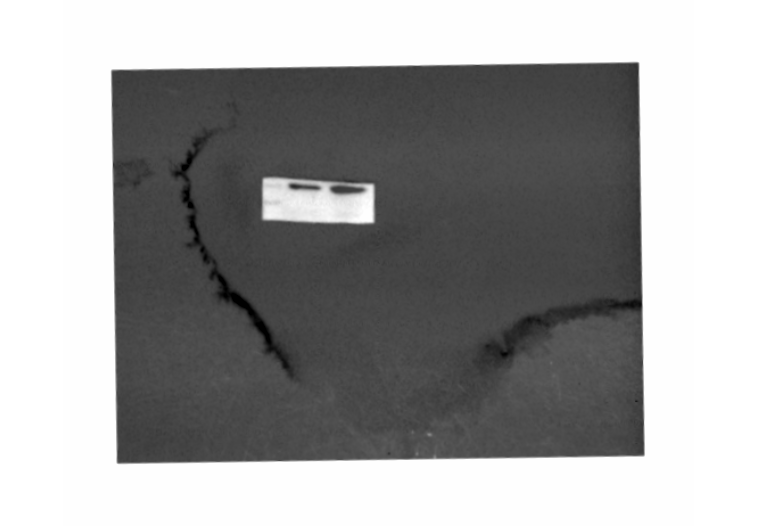


N4BP1


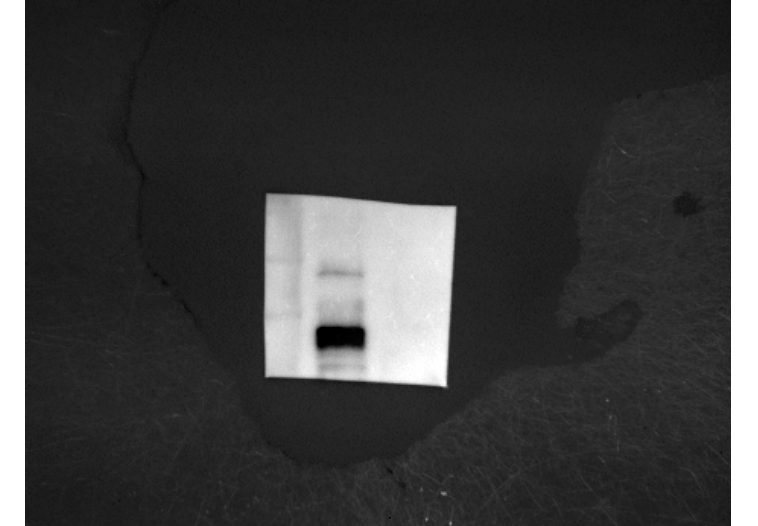


GAPDH


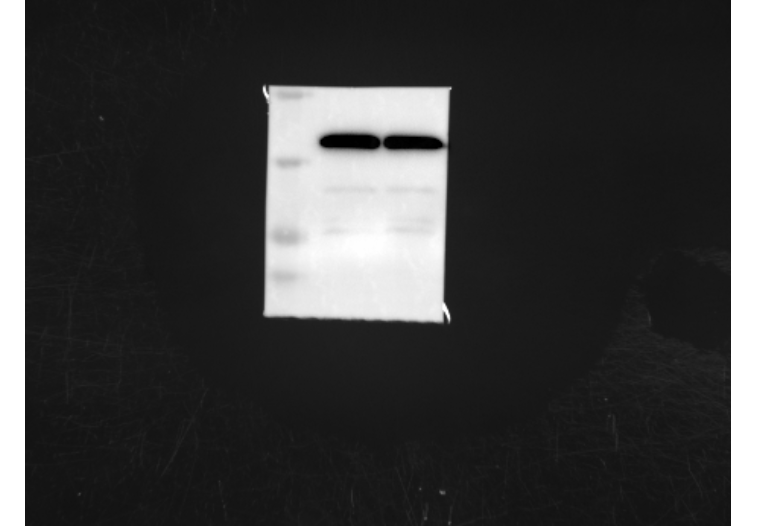


Fig S4C

CXCL8


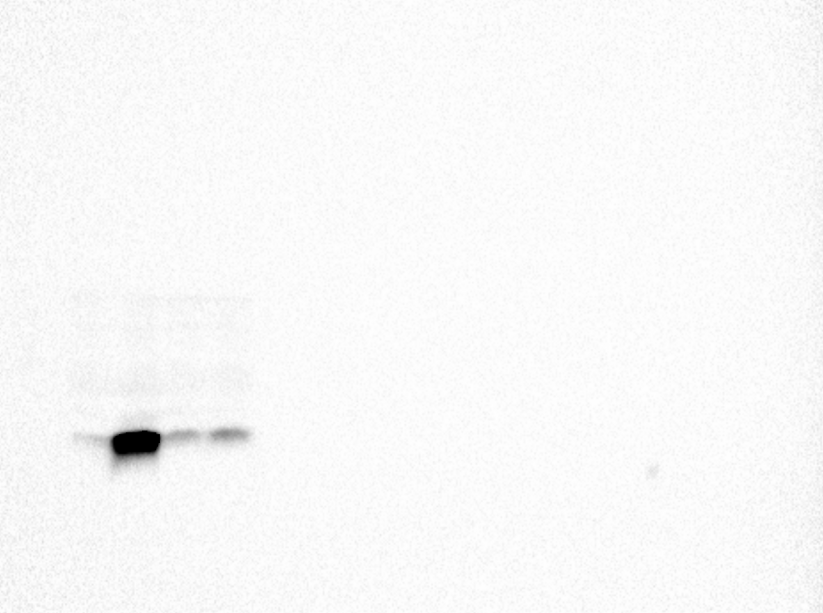


FLAG-N4BP1


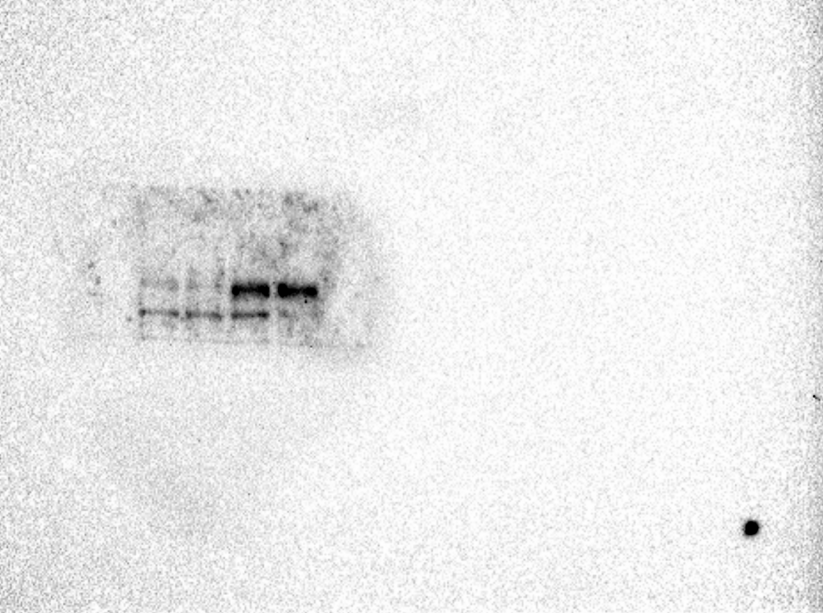


GAPDH


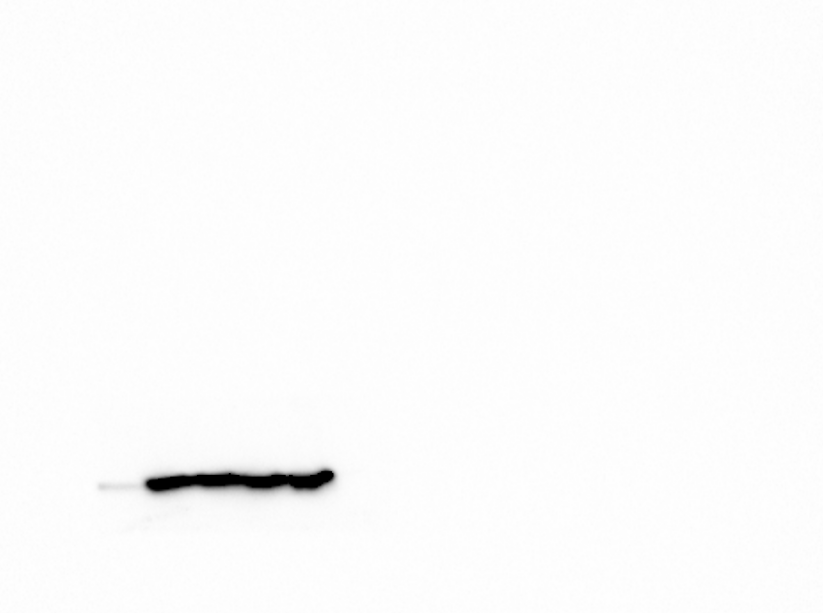


Fig S4F

SCC9-S100A2


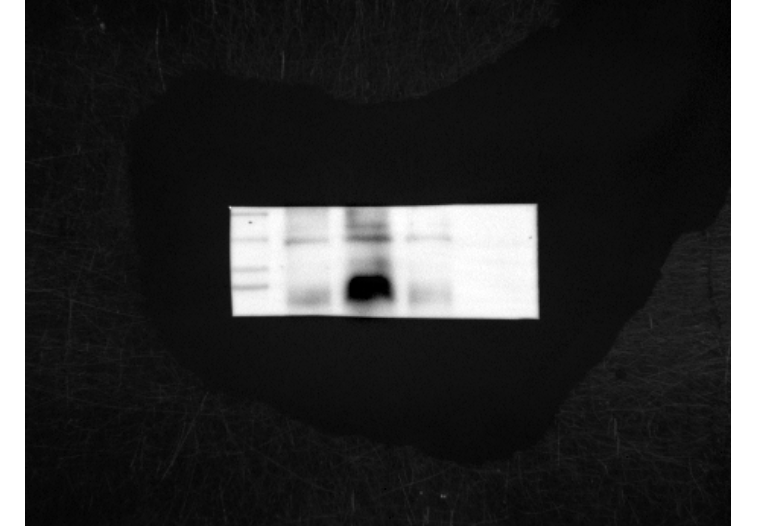


SCC9-N4BP1


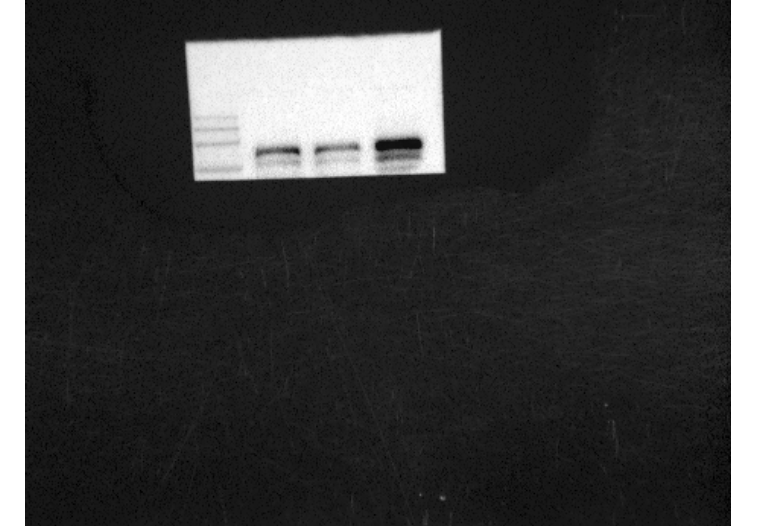


SCC9-HSP70


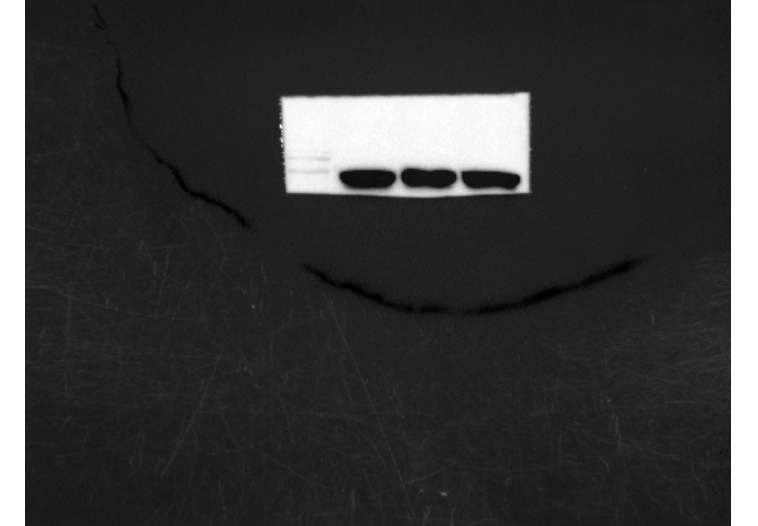


CAL27-S100A2


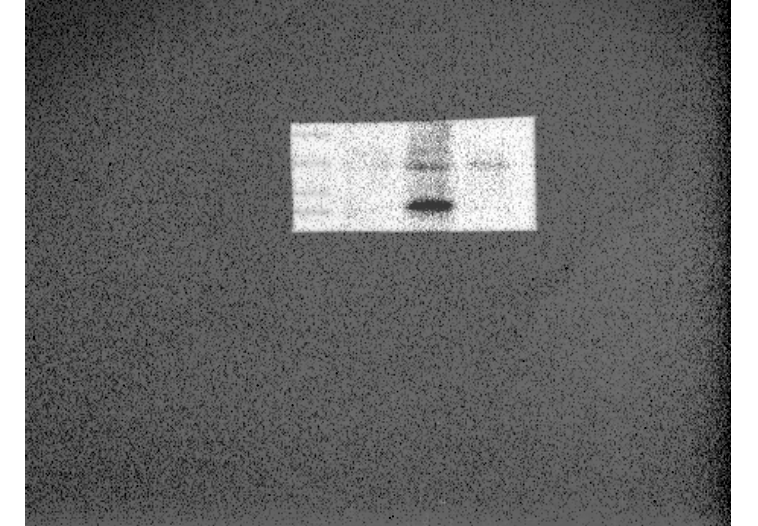


CAL27-N4BP1


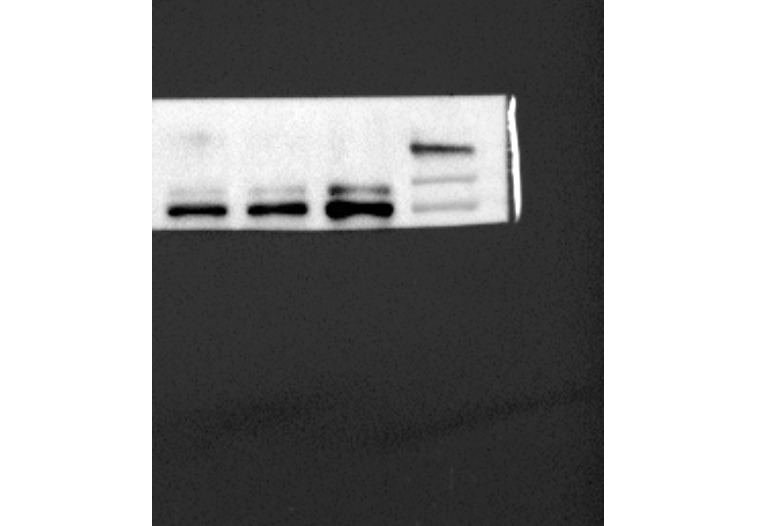


CAL27-HSP70


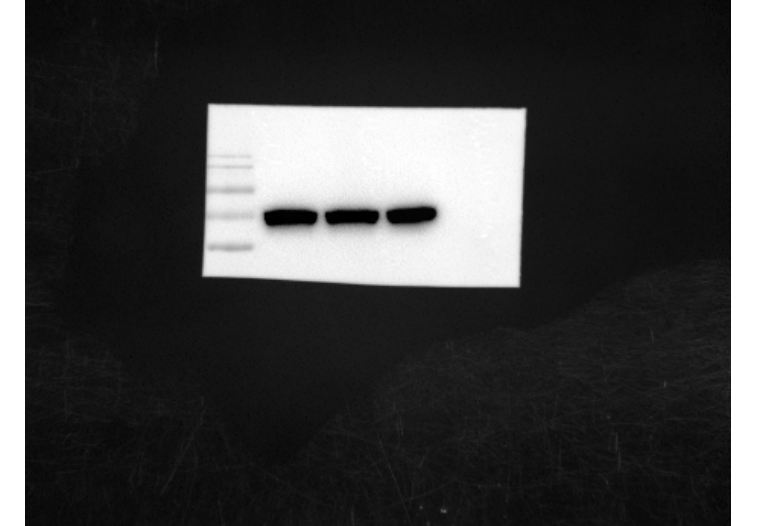


Fig S4G

SCC9-S100A2


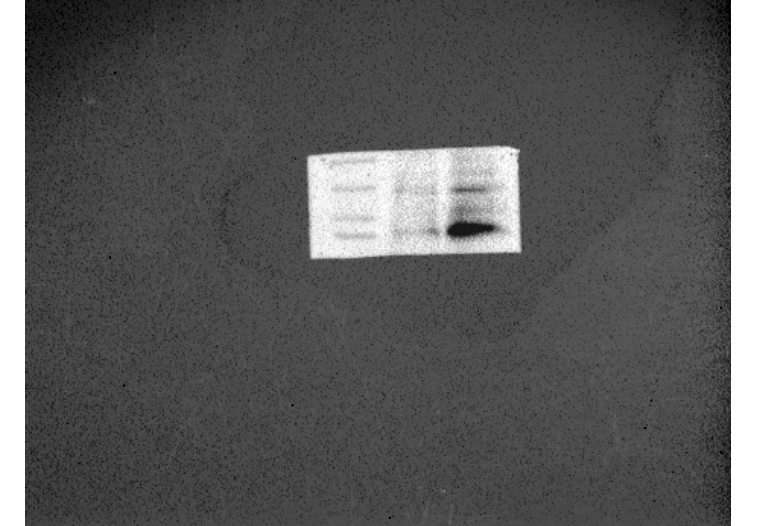


SCC9-HSP70


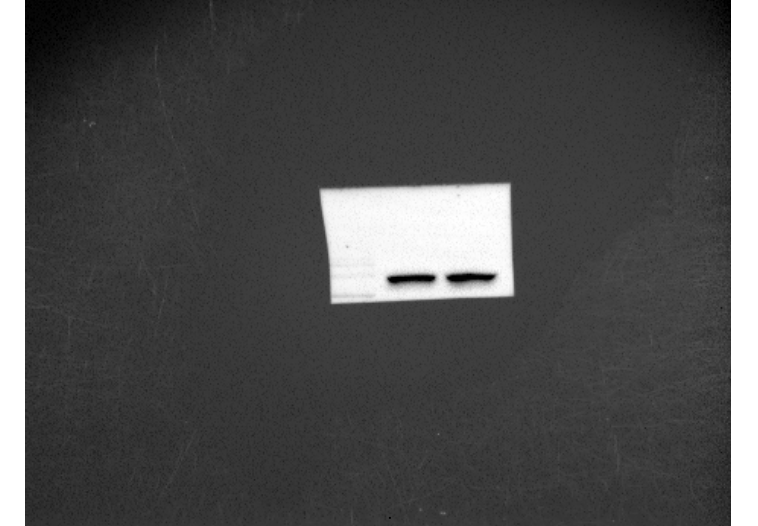


CAL27-S100A2


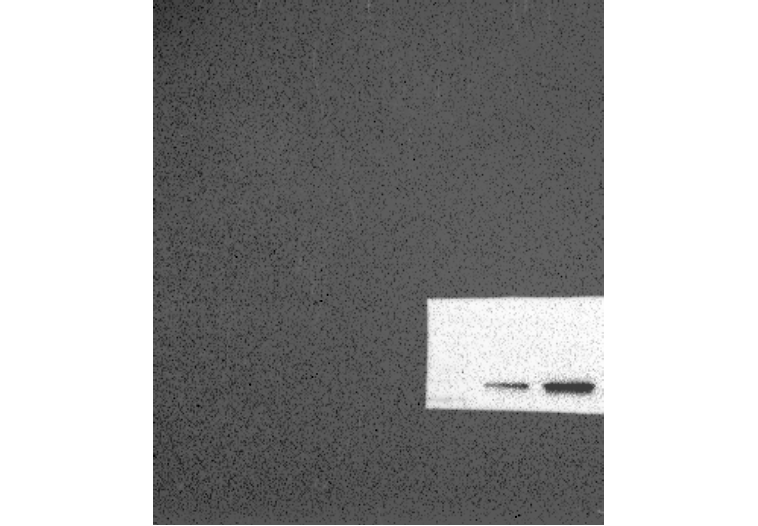


CAL27-GAPDH


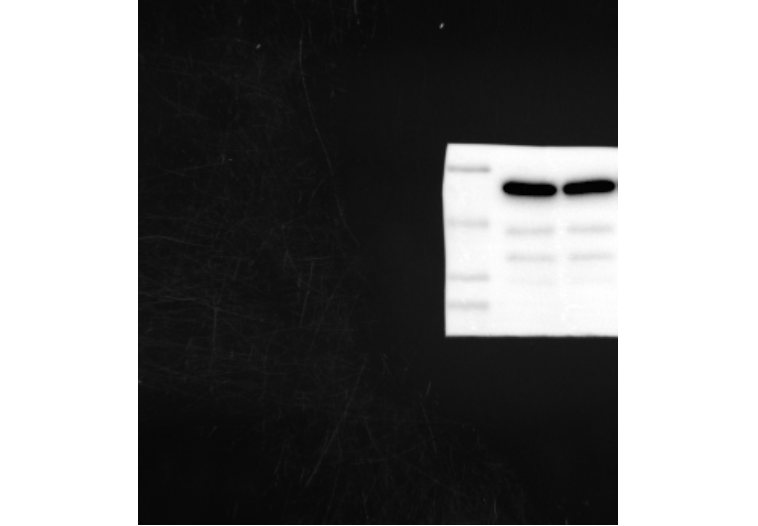

Supplement: Supplementary file 9 — Original WB Images [file 41419_2025_8229_MOESM9_ESM.docx]
